# Low temperature synthesis of NbC/C nano-composites as visible light photoactive catalyst

**DOI:** 10.1038/s41598-018-31989-z

**Published:** 2018-09-11

**Authors:** Aayush Gupta, Manish Mittal, Mahesh Kumar Singh, Steven L. Suib, Om Prakash Pandey

**Affiliations:** 1 School of Physics and Materials Science, Thapar Institute of Engineering and Technology, Patiala, 147004 India; 2 Department of Mechanical Engineering, Indian Institute of Science, Bangalore, 560012 India; 3 Department of Chemistry, University of Connecticut, 55 North Eagleville Rd., Storrs, Connecticut 06269 USA

## Abstract

A facile carbothermal route was adopted to obtain niobium carbide nanoparticles (NPs) embedded in carbon network from Nb_2_O_5_ to study photocatalytic behavior. Optimization of synthesis parameters to obtain single phase NbC NPs has been successfully done. The phase identification, morphology and nature of carbon were determined with the help of X-ray diffraction, transmission electron microscopy (TEM) and Raman spectroscopy. X-ray photoelectron spectroscopy (XPS) suggested the presence of multiple oxidation states of Nb associated to NbC and NbC_x_O_y_ centers on the surface of NPs. Due to the presence of NbC_x_O_y_ on the surface of NPs, absorption under visible region of EM spectrum has been observed by UV-visible spectroscopy. Different organic dyes (RhB, MB and MO) were used to study the effect of holding time on the photocatalytic performance of as-synthesized samples. RhB dye was found to be the most sensitive organic molecule among all the considered dyes and degraded 78% in 120 min.

## Introduction

The extensive spillage of organic pollutants in water from industries has led to severe environmental problems. These organic pollutants are not only creating aquatic pollution, but also disturbing the ecological balance. The required biochemical oxygen demand (BOD) in river streams of most Asian rivers has decreased due to organic pollutants released by industries^1,2^. Moreover, dyes are colored and cause hindrance to the sunlight penetration and reduction in dissolved oxygen, which affects aquatic living organisms^3^. About 15% of industrial dyes are being disposed without any treatment^4^. Among different types of treatment methods (ultrafiltration, chemical oxidation, biosorption etc.), photocatalysis has emerged as a green technology due to the complete mineralization of pollutants to water, CO_2_, and mineral acids even at room temperature^5,6^.

Metal oxides viz. TiO_2_, ZnO and carbon allotropes (graphene materials) are the common photocatalysts used for degradation of different dyes under UV-visible irradiation^7–13^. Dissimilar to these photocatalysts, metallic photoconductors contain either small band gaps or absence of energy difference between highest occupied and lowest unoccupied bands^14^. Metallic photocatalysts exhibit the generation of electron-hole pair due to inter-band transitions (fully occupied conduction band to partially occupied conduction band) and higher carrier density than semiconducting photocatalysts make them suitable as photocatalysts^15^. Transition metal carbides (TMCs), excellent high-temperature stable compounds exhibiting high corrosion and tribological properties, are now being exploited for other applications like catalyst supports in electrochemical cells^16–19^. Among all the TMCs, niobium carbide (NbC) is a highly stable compound and is mostly used in nuclear and thermal engines^20–22^. Different morphology like thin films, micro-cage shaped, nano wires and fibers of NbC have been synthesized to extract the required properties suitable for different applications such as corrosion resistance, catalyst supports, rotors of micro ceramics engines, conduction and electrodes for electrochemical cells, respectively^16,23–27^. As a function of microstructure, Coy *et al*.^28^ studied the electrocatalytic activity of NbC thin films for hydrogen evolution reaction (HER). Further, NbC thin film has also been found as a electrocatalyst support for both alkaline and acidic mediums for HERs^29^. Carbon coated TMCs have drawn the attention of researchers for their unique physicochemical properties for electrocatalytic H_2_ production, targeted drug delivery, flexible electronics, magnetic properties and electro-oxidation^30–34^. Encapsulation of NPs help to overcome the agglomeration and oxidation limitations particularly for high temperature applications of nano TMCs. Among all the elements, carbon is preferred for encapsulation due to good electrical conductivity and stability in different chemical environments and has a high specific surface area in different forms^24,35,36^. Carbon as coating hinders the oxidation of NPs as reported by Wang *et al*.^37^ and Singla *et al*.^32^ for tungsten carbide NPs which enables these nanomaterials to be used in various electro oxidation reactions^38^.

Various researchers have practiced encapsulation of carbon on NPs through different synthesis techniques and chemical reagents^24,25,39–47^. To overcome the high temperature and time consuming procedures, carbothermal synthesis in an autoclave has been practiced at low temperature^32,34^. With the help of an autoclave, various reactants were subjected to carburization of the transition metal (V, Co, W, Ti) source in the presence of a reducing agent to obtain nano TMCs at relatively low temperature^30,32,34,48^.

Although a significant amount of work has been reported on the synthesis of carbon encapsulated TMCs^30,32–34,41,49,50^, the developing process is not so economical. Zhang *et al*.^42^ dsynthesized core-shell NbC/C nano spheres by laser ablation methods in which they used a Nb target in a carbon rich liquid environment (ethanol) for ablation. As far as photocatalytic applications are concerned, except for WC and Mo_2_C, other carbides are less studied^51,52^. Chen *et al*.^52^ have reported the photocatalytic degradation of RhB with the help of NbC in almost 12 h under a 300 W mercury lamp. A detailed analysis of photocatalytic characteristics of NbC and coated NbC is not available. In the present approach, NbC/C nano-composite has been synthesized via a carbothermal route. A mechanism responsible for the formation of NbC NPs has been proposed from the data obtained by various crystallographic, thermal, microstructural, spectroscopic, and thermodynamic calculations. The photo-enhanced catalytic efficiency of NbC/C nano-composite has been studied by using RhB, MB, and MO dye organic model compounds, frequently used as a tracer, bright colorant of wool, Nylon fabric, metal detection, and as a food colorant^53,54^.

## Results and Discussion

### XRD analysis

**Figure 1:**
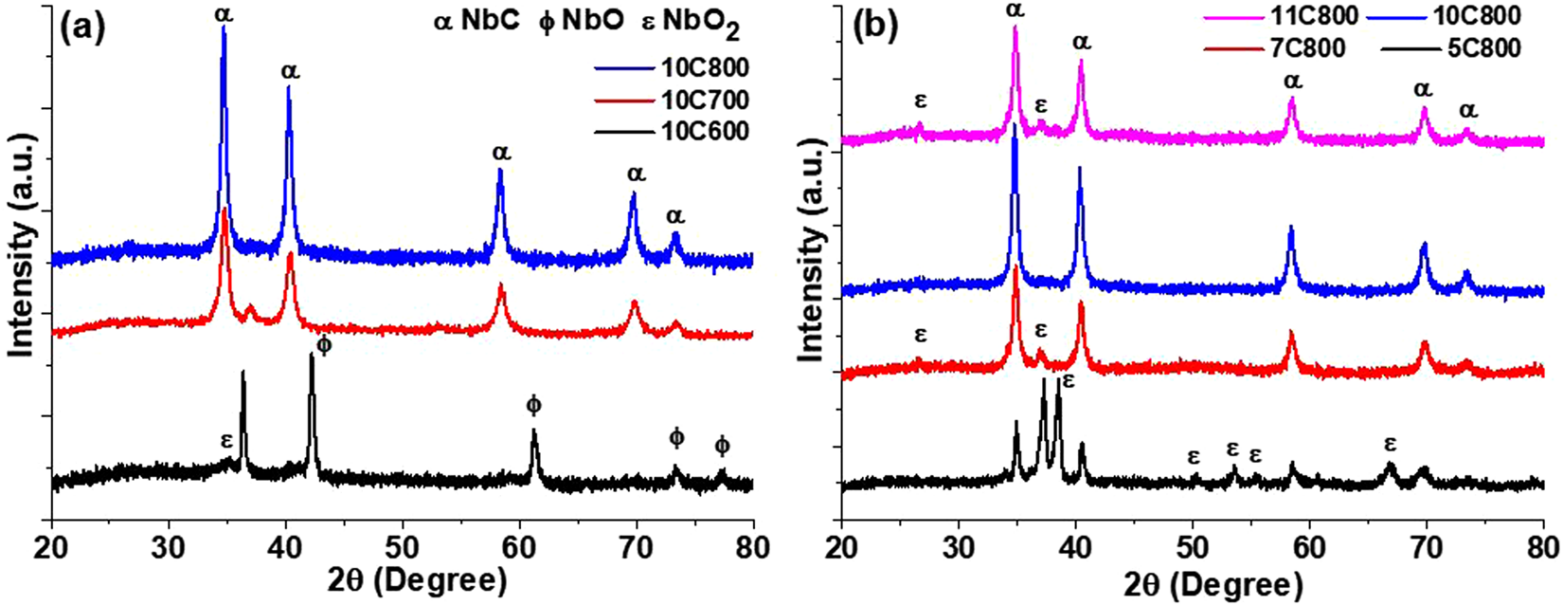
X-ray patterns of all the synthesized samples (**a**) at different temperatures with holding time of 10 h and (**b**) at 800 °C with different holding time of 5, 7, 10 and 11 h.

For the synthesis of single phase carbon coated NbC NPs, synthesis temperatures and holding times were optimized on the basis of XRD results which are shown in Fig. 1a,b respectively. In both figures, peaks marked with ‘α’ depict the rock salt structure (space group Fm-3m) NbC which were matched with (ICDD 01-089-3690). The relative carbon content of synthesized NbC samples was also calculated by a = 4.09847 + 0.7128x − 0.3457x^2^ where a = lattice parameter in Å and x = atomic ratio C/Nb and listed in Table 1^55^. For the optimization of synthesis parameters (temperature and holding time) single phase NbC NPs were synthesized using experiments discussed below.

**Table 1:** Details of lattice parameter and carbon content of NbC in different synthesized samples.

Sample label	Temp. (°C)	Holding time (h)	Lattice parameter (Å)	C- content (x)
10C600	600	10	—	—
10C700	700	10	4.4620	0.874
10C800	800	10	4.4692	0.959
5C800	800	5	4.4576	0.799
7C800	800	7	4.4639	0.902
11C800	800	11	4.4650	0.904

### Optimization of synthesis temperature

XRD patterns of samples synthesized at low temperatures (600, 700 and 800 °C) with 10 h holding is shown in Fig. 1a. The synthesis at 600 °C results in the reduction of Nb_2_O_5_ to NbO (sample 10C600). While, at 700 and 800 °C, mixtures of (NbC + NbO_2_) and NbC were obtained respectively. The absence of the NbC phase at 600 °C (Fig. 1a) may be due to the unavailability of enough thermal energy to further reduce and carburize NbO to transform into NbC which can be seen in the case of 10C600. Sample 10C700 shows the conversion of Nb_2_O_5_ to NbC with little NbO_2_ showing a reduction and carburization path. A further increase by 100 °C (sample 10C800) resulted in the complete transformation of Nb_2_O_5_ to NbC without showing any signature of lower oxides of Nb.

### Optimization of holding time

After obtaining single phase NbC at 800 °C, the holding time has been varied to study the behavior of reduction and carburization of Nb_2_O_5_ with optimized temperature which is shown in Fig. 1b. Sample 5C800 shows a reduction of Nb_2_O_5_ after carburization and the formation of a mixture of NbO_2_ and NbC. Sample 7C800 showed contrary results in which carburization exceeded the reduction process as shown by NbO_2_ as a minor phase and NbC as a major phase. In the case of 10 h, complete reduction followed by carburization of Nb_2_O_5_ was observed in sample 10C800 (Fig. 1b) resulting in single phase NbC. Furthermore, the increment of holding time to 11 h led to decarburization of NbC with the formation of NbO_2_. The decarburization of NbC may be caused by a lower CO/CO_2_ ratio (CO_2_ rich reaction) beyond 10 h at 800 °C^56,57^. While the formation of NbO_2_ as a result of decarburization at longer holding (11 h) instead of NbO (as observed in the case of lower temperature) may be attributed to the higher stability of NbO_2_ than NbO^58^.

Optimization of synthesis parameters to obtain single phase NbC also affects the constituted crystallographic distortion. In order to understand all the distortional transitions that occurred in NbC due to different synthesis temperatures and holding times, stress-strain analysis (Williamson-Hall) of the obtained samples has been done. Detailed theory and assumptions of Williamson-Hall analysis has been provided in Appendix-II.

### Williamson-Hall analysis

Table 2 depicts a comparative study of all the postulates of W-H analysis and Scherrer criteria for all the synthesized NbC samples. The least magnitude of strain in NbC lattice at higher synthesis temperature (sample 10C800) is very obvious due to complete conversion of Nb_2_O_5_ to NbC, while presence of NbO_2_ at 700 °C (sample 10C700) distorts the crystallites which shows higher strain in crystallites. As a function of holding time at 800 °C, the induced strain tends to decrease as carburization occurs. Figure 1b shows that up to 10 h of holding, minor phases are being eliminated (5 h (NbC + NbO_2_) to 7 h (NbC + NbO_2_) then 10 h (pure NbC)) leading to a large reduction in strain. The diffusion of carbon in the Nb lattice is increased by increasing the holding time which supports the enhancement of lattice parameters by reducing the strain and supporting the attainment of equilibrium positions of Nb and C atoms in the NbC unit cell. Beyond 10 h (11C800), decarburization of NbC led to increase the distortion which might be associated to incorporation of oxygen resulting NbO_2_.

**Table 2:** Williamson-Hall Analysis.

	USM		USDM			USEDM				Scherrer Method (nm)
	ε × 10^−4^	t (nm)	ε × 10^−4^	σ (GPa)	t (nm)	ε × 10^−4^	σ (GPa)	u × 10^−5^ (kJm^−3^)	t (nm)	
10C700	19.5	16.7	13.2	0.56	14.8	0.4	0.02	56.7	15.7	10.8
5C800	85.1	27.6	327.4	2.79	77.8	8.9	0.38	1296	36.8	18.3
7C800	31.8	33.7	26.2	1.10	27.4	18.5	0.05	185.0	31.1	13.6
10C800	18.5	25.7	12.9	0.54	22.0	0.3	0.01	52.6	23.8	14.9
11C800	30.2	33.7	123.8	1.04	27.8	1.1	0.05	165.8	31.1	14.0

### XPS analysis

**Figure 2:**
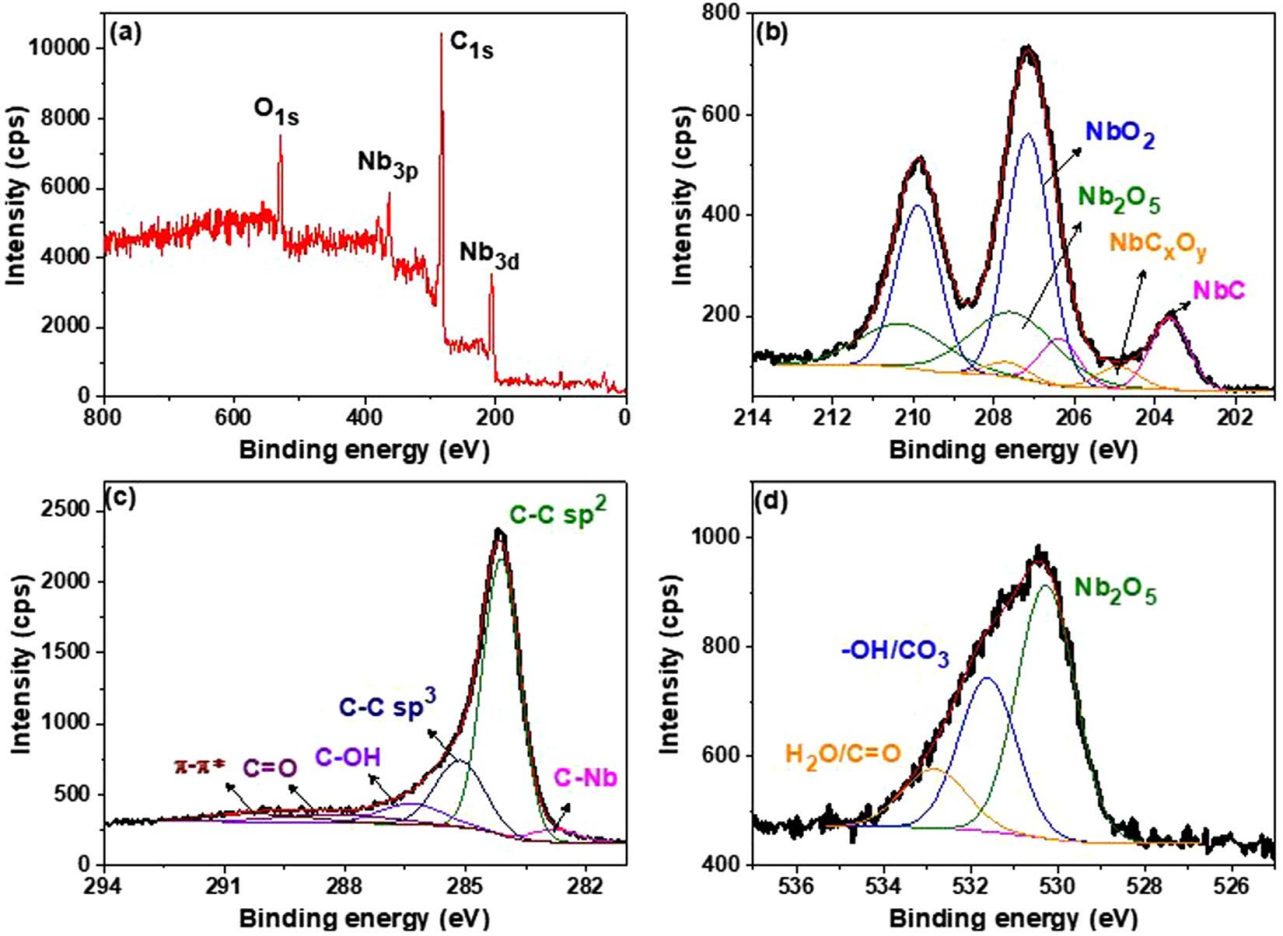
XPS spectra of sample 10C800: (**a**) survey; (**b**) HR-XPS of Nb 3d; (**c**) HR-XPS of C 1 s and (**d**) HR-XPS of O 1 s.

XPS analysis of all the samples synthesized at 800 °C was carried out to understand the surface chemical variations (valence states) that reactants undergo during reduction and carburization processes. The survey spectrum confirms the presence of Nb, C and O on the surface of as-synthesized samples which is shown in Figs 2a and S1. High resolution XPS (HR-XPS) spectra of Nb3d, C1s and O1s transitions are shown in Figs 2(b–d) and S1 respectively in which different chemical states of Nb, C, and O have been illustrated. Peak positions and relative contents of all the elements in various valence states have been listed in Table 3. The convoluted HR-XPS spectrum of Nb 3d is composed of doublets corresponding to spin orbital splitting of 3d_5/2_ and 3d_3/2_ as shown in Figs 2b and S1.

**Table 3:** Position and area under the de-convoluted peaks of different elements observed in high resolution XPS spectra of 5C800, 7C800, 10C800 and 11C800.

Element	Group	FWHM (eV)	5C800		7C800		10C800		11C800		Ref.
			Position (eV)	Vol. fract. (%)	Position (eV)	Vol. fract. (%)	Position (eV)	Vol. fract. (%)	Position (eV)	Vol. fract. (%)	
Nb_3d_	NbC	1.23	203.65	4.66	203.60	2.90	203.63	13.72	203.67	7.17	^59, 60^
			206.39		206.34		206.37		206.41		
	NbC_x_O_y_	1.21	—	—	204.72	2.65	204.91	4.54	204.94	2.09	
			—		207.46		207.65		207.68		
	NbO_2_	1.26	207.25	67.84	206.90	55.32	207.14	53.44	206.83	64.58	
			209.99		209.64		209.88		209.57		
	Nb_2_O_5_	2.51	207.58	27.50	207.37	39.13	207.52	28.28	207.54	26.16	
			210.32		210.11		210.26		210.28		
	**Nb content (%)**		**8.49**		**11.74**		**11.54**		**10.38**		
C_1s_	C-Nb	1.35	282.85	2.15	282.81	2.91	282.79	3.35	282.78	3.44	^60, 61^
	C sp.^2^	1.08	284.40	56.10	284.40	54.10	284.40	59.39	284.40	51.60	^59, 62^
	C sp.^3^	1.43	285.01	18.72	285.08	23.57	285.13	18.99	284.92	25.84	^62, 63^
	C-OH	1.87	286.01	12.99	286.46	4.50	286.32	7.72	286.40	7.80	
	COOH	3.32	288.22	5.49	288.02	10.22	288.17	5.51	288.29	4.83	
	π-π*	3.11	290.18	4.57	289.71	4.70	290.13	5.01	290.39	6.49	
	**C content (%)**		**78.25**		**72.65**		**76.15**		**75.17**		
O_1s_	Nb_2_O_5_	1.56	530.43	53.79	529.98	42.49	530.26	53.68	529.98	49.48	^64^
	OH/CO_3_/O_v_	1.54	531.79	25.35	531.32	39.85	531.60	32.23	531.35	24.24	^64^
	H_2_O/C=O	1.76	532.84	20.87	532.52	17.67	532.83	14.07	532.29	26.28	^65^
	**O content (%)**		**13.26**		**15.61**		**12.31**		**14.45**		

Figure 2b revealed the presence of multiple oxidation states of Nb associated to different compounds (NbC, NbC_x_O_y_, NbO_2_ and Nb_2_O_5_) even after obtaining single phase XRD pattern of 10C800 as shown in Fig. 1a. Such HR-XPS spectra of single phase NbC (10C800) suggests the generation of different ionic states of Nb during the reduction-carburization process. Variation in the holding time at 800 °C, led to change the chemical composition of samples (suggested by XRD results) which can also be observed in XPS results as shown in Fig. S1. Volume fraction (VF) of different phase present on the surface has also been calculated by deconvoluting the XPS spectrum and listed in Table 3. Further, the increment in the holding time led to enhance carburization up to 10 h as VF of NbC has increased (4.66% to 13.72%) and decreased in 11C800 (7.17%) supporting the XRD results. VF of Nb^4+^ (NbO_2_) and Nb^5+^ (Nb_2_O_5_) has increased (7C800; 94.45% and 11C800; 90.74%) as the holding time was shifted from 10 h (81.72%). Moreover, binding energy associated to NbC_x_O_y_ (~204.8 eV) was not observed in 5C800. HR-XPS data for Nb3d suggest that direct conversion of Nb_2_O_5_ to NbC involved reduction and carburization simultaneously which was responsible for the multiple transitions of oxidation states of Nb in the lattice as earlier discussed for XRD analyses. The HR-XPS spectrum of C 1 s as shown in Figs 2c and S2 revealed the presence of peaks associated with NbC, C-C sp^2^ and C-C sp^3^ bonds in all the samples at 282.8, 284.4 and 285.1 eV, respectively^59–63^. Further, peaks observed around 286.4, 288.1 and 290.1 eV might be associated to hydroxyl, carbonyl groups and π → π* transitions, respectively^62,63^. HR-XPS spectra of O 1 s (Figs 2d and S1) suggested the presence of oxygen associated to Nb_2_O_5_ at 530.4 eV. While, the peaks around 531.6 and 532.5 eV may be associated to hydroxyl group/carbonate species/oxygen vacancies (O_v_) and adsorbed water/carbonyl (C=O) group, respectively^64,65^.

### Microstructural analysis

**Figure 3:**
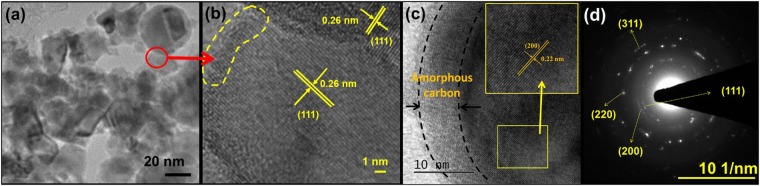
(**a**) TEM micrograph of 10C800 showing agglomeration of core shell structure of carbon coated NbC NPs; (**b**) HR microstructure of marked circle in 4(a) showing inter-planar spacing of plane (111) of NbC, (**c**) HR micrograph of NbC NP showing carbon coating (marked with black dotted region) on NbC particle and inset shows the lattice fringes corresponding to (200) plane; (**d**) selected area electron diffraction pattern of agglomerated NbC NPs (shown in 4a) depicting poly-crystallinity.

TEM micrographs of 10C800 are shown in Figs 3 and S2 showing the agglomerated NPs which is attributed to their nano regime. Agglomerated NbC NPs of 10–30 nm are encapsulated in carbon network as shown in Figs 3a and S2. Figure 3b,c show the lattice fringes corresponding to (111) and (200) planes having inter-planar spacing of 0.26 nm and 0.22 nm, respectively for NbC. The indistinct interface between the NbC (core) and the amorphous carbon layer is attributed to the solid state diffusion of carbon as shown in Fig. 3b,c (marked with dotted lines at the periphery of the particle). Figure 3d shows the selected area electron diffraction pattern of the area shown in Fig. 3a, indicating nano crystallinity by concentric diffraction spots corresponding to various atomic planes. Furthermore, a narrow particle size distribution was observed having 12 nm as average particle size of as-synthesized sample as shown in Fig. S3.

**Figure 4:**
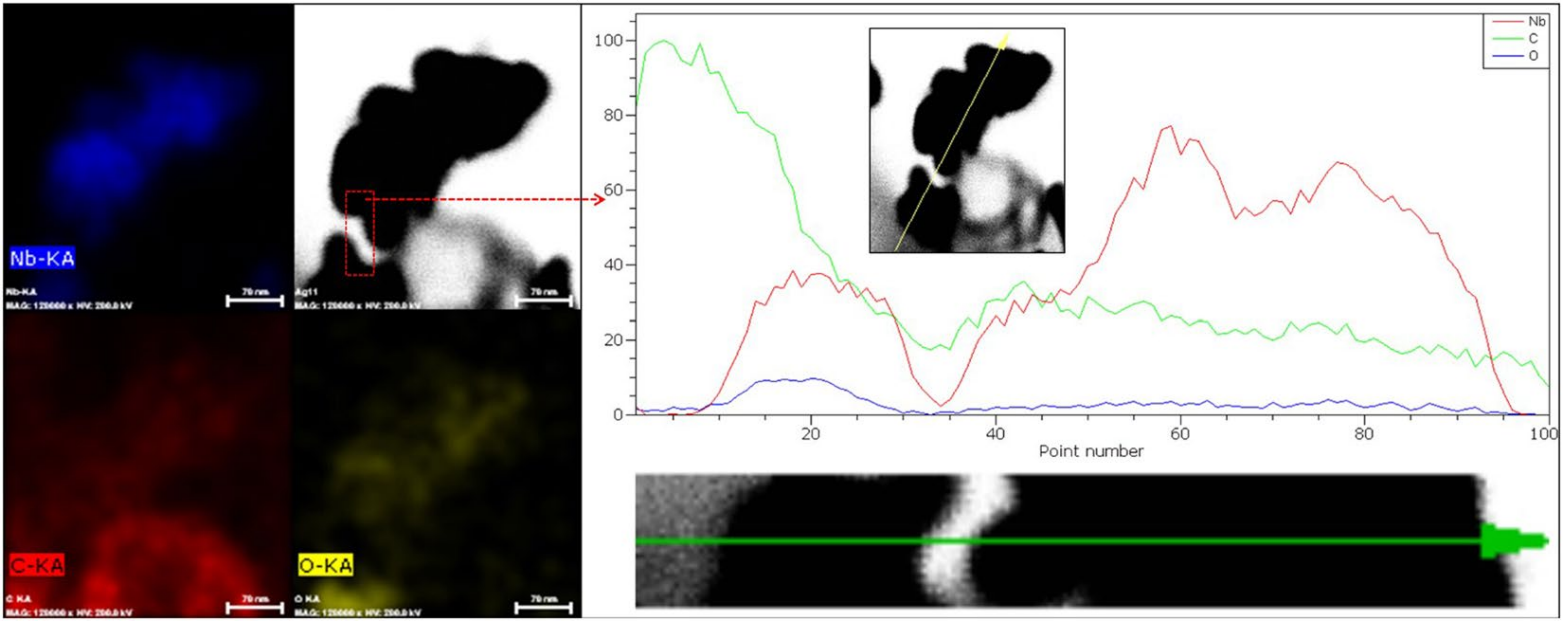
Elemental line profile of NbC nanoparticle (10C800) showing the concentration profile (Nb, C and O) across the shown nanoparticles (marked with green arrow). Elemental line profile suggests the homogenous distribution of oxygen inside the NbC nanoparticles while, smaller agglomerate contain higher O content than larger agglomerate.

Further, to observe the distribution of Nb, C and O in the synthesized NbC NPs (10C800), elemental profile was also carried out as shown in Fig. 4 in which distribution of carbon is nearly same as carbon is also present as amorphous network. While, concentration of oxygen is relatively low throughout the particle confirming the presence of oxygen centers in the NbC nanoparticle resulting NbO_x_ or NbC_x_O_y_. Further, elemental line profile (STEM) was also taken to observe the linear distribution of each elements from periphery to core which is also shown in Fig. 4. Extreme left region of line profile shows the higher C content on the left side of nanoparticle which may be associated to the amorphous carbon network. Moreover, TEM micrograph of considered region for EDAX analysis is shown in Fig. S2 and the presence of Nb, C and O were confirmed along with Cu (unmarked peak at 8 keV) which is associated to grid. On the nanoparticle region marked by arrow (green colored), increase in the concentration of Nb (red) and C (green) is more as compared to O (blue) confirming the presence of oxygen centers in NbC nanoparticles. Such concentration profile of Nb and O throughout the nanoparticle suggest the presence of multiple oxidation states of Nb corresponding to NbC and NbC_x_O_y_ (NbO_x_ centers) as observed from XPS analysis.

### Surface area analysis

**Figure 5:**
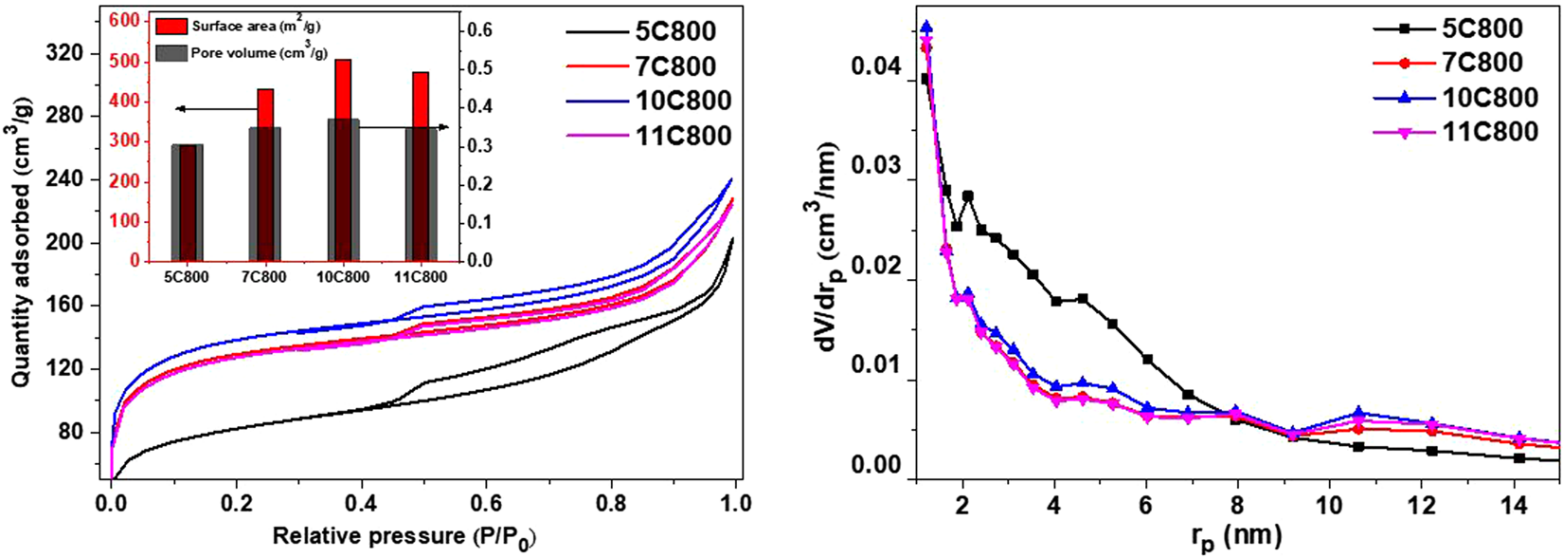
BET analysis of all the samples synthesized at 800 °C.

BET specific surface areas (SSA) were calculated from adsorption-desorption curves using N_2_ gas for the samples synthesized at 800 °C as shown in Fig. 5. BET analysis suggests the highest SSA of 506 m^2^/g with 0.3697 cm^3^/g pore volume for 10C800 while, the presence of oxide and Nb metal with NbC in other samples induced the reduction in SSA and pore volume as shown in the inset of Fig. 5, also observed by Gupta *et al*.^49^. Such variation of SSA and pore volume of synthesized samples might have occurred due to evolution of CO or CO_2_ as a result of *in situ* reduction and carburization of niobium oxide by encapsulated carbon. Further, pore size distribution suggested the contribution of high surface area and mesopores of as-synthesized samples as shown in Fig. 5.

### Proposed synthesis mechanism

Normally NbC is synthesized at >1000 °C^23,25,66^ which is higher as compared to the present work. The transition of Nb_2_O_5_ to NbC at relatively low temperature (800 °C) in a high pressure closed chamber is a temperature driven transition. The mixture of reactants contains Mg and charcoal, which act as reducing agents providing MgO, CO and CO_2_ as byproducts. The gaseous byproducts (CO and CO_2_) of the reactions lead to increase the pressure inside the autoclave. Various chemical reactions occurring inside the autoclave have been listed in the supplementary information.

**Figure 6:**
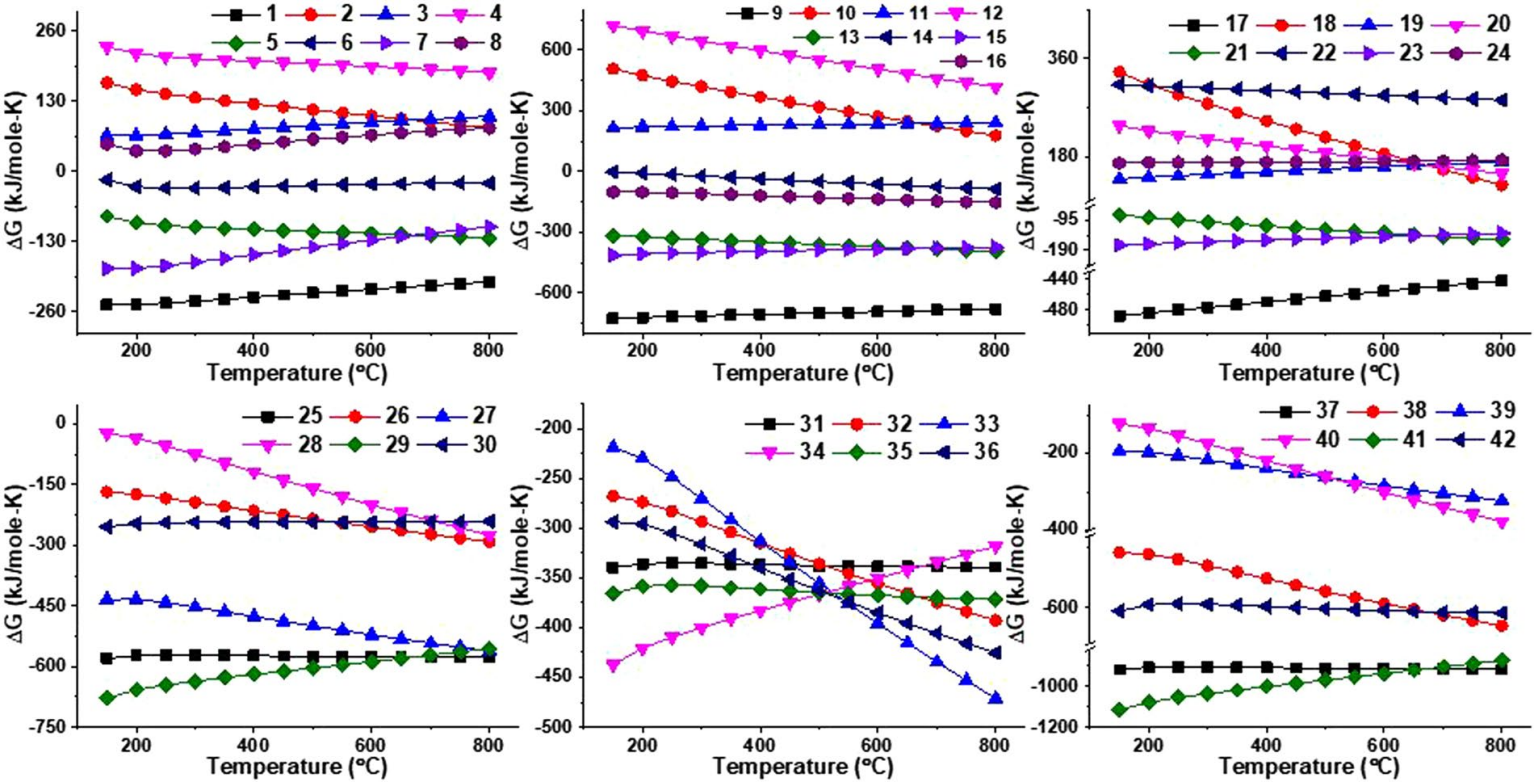
Feasibility and non-feasibility of possible reaction paths to form NbC from Nb_2_O_5_ via multi-step route.

All the possible chemical reactions are categorized into two categories; (i) reduction and (ii) carburization. Reactions (S1–S24) represents the reduction of Nb_2_O_5_ forming NbO_2_, NbO and (NbO_2_ + NbO) mixture in the presence of reducing agents (Mg and C) and their mixture. All the reduction reactions suggested that Mg (alone) reduced Nb_2_O_5_ more efficiently to obtain NbO_2_, NbO and (NbO_2_ + NbO) mixture as compared to C and (C + Mg) mixture as represented by reaction (S1, S9, S17) in Fig. 6. Further, C and CO (in the presence of Mg) reduced Nb_2_O_5_ equivalently to obtain similar products as reaction (S5, S13, S21) and (S7, S15, S23), respectively, which are shown in Fig. 6. Rest of the other reduction reactions are non-feasible due to their positive ΔG suggesting that individually C and CO (in the absence of Mg) are not capable enough to reduce Nb_2_O_5_.

As a result of reduction reaction, feasibility of the formation of NbO is higher than that of NbO_2_ (more negative ΔG of reaction S9, S17 than S1) which is also supported by the XRD pattern of sample 10C600. Further, the formation of NbC can be obtained via *in-situ* reduction carburization of oxide products (NbO_2_ and NbO) as Nb metal was not obtained in any of the synthesized sample (as shown in Fig. 1). In the similar pattern, reaction (S25–S42) represent the possible chemical reactions for the formation of NbC from NbO_2_, NbO and (NbO_2_ + NbO) mixture, all are feasible with negative ΔG values. Unlike reduction reactions, reduction-carburization of NbO_2_ and NbO follow different paths as shown in Fig. 6 by reaction (S25–S30) and (S31–S36), respectively. While, reduction-carburization of (NbO_2_ + NbO) mixture is represented by reaction (S37–S42) which are more spontaneous than previous chemical reactions (S25–S36) with high difference of ΔG values. As single phase NbC was obtained at 800 °C, reduction-carburization reactions follow the following order according to ΔG at 800 °C; S37 > S41 > S38 > S42 > S25 > S27 > S29 > S33 > S36 > S32 > S40 > S35 > S31 > S39 > S34 > S26 > S28 > S30.

**Figure 7:**
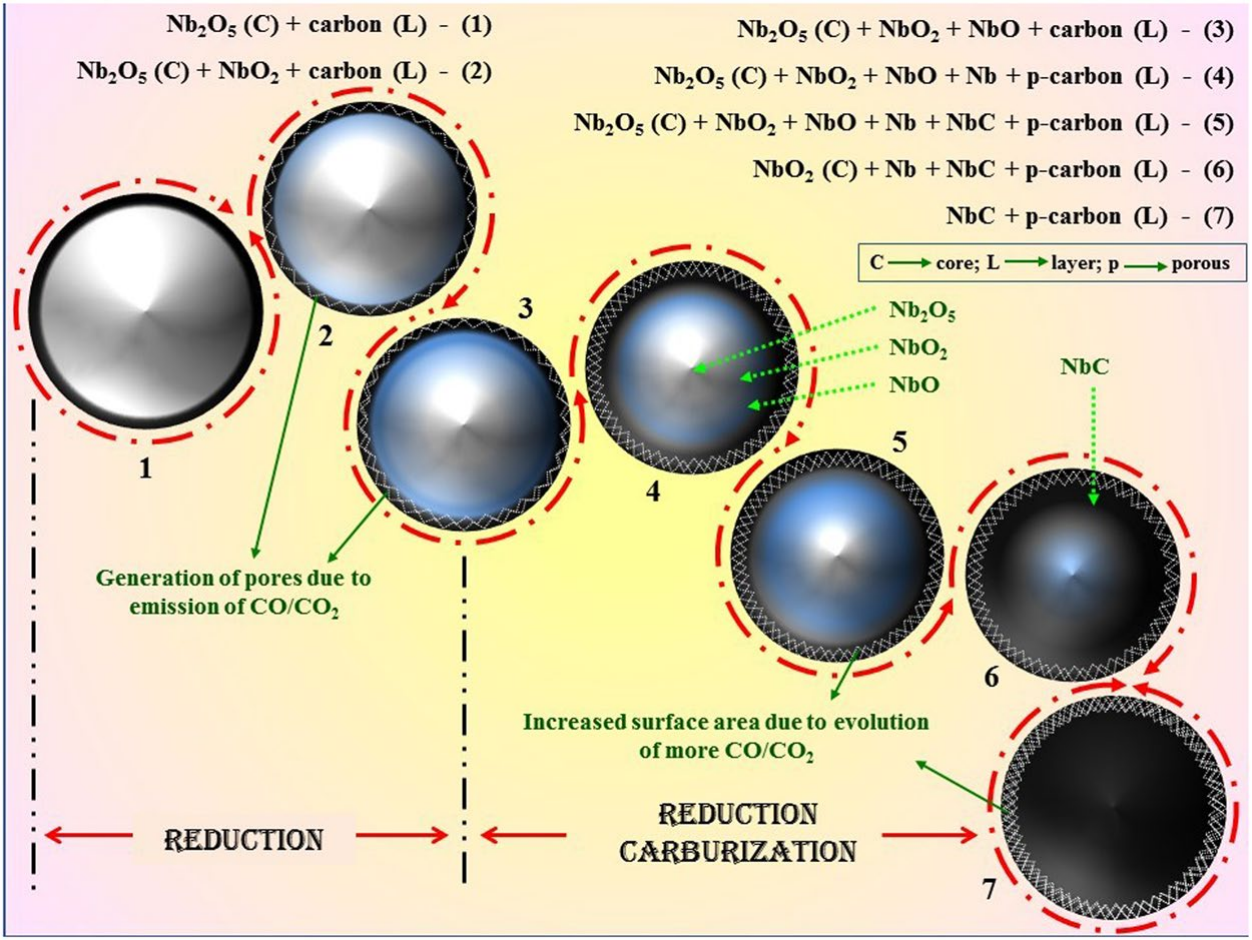
Reaction mechanism of the formation of NbC using charcoal as carbon source with Nb_2_O_5_.

Thermodynamically higher feasibility of the formation of NbO_2_ + NbO mixture is also evidenced in the XRD pattern (Fig. 1a) of 10C600 where NbO (major) + NbO_2_ (minor) were obtained at 600 °C. This is further converted to NbC (major) + NbO_2_ (minor) at 700 °C and NbC at 800 °C. In this sequence, charcoal encapsulated NbO particles which is followed by reduction-carburization reactions. As a result, CO and CO_2_ gases evolved through coated carbon resulting to porous particles. Moreover, the transition $(N{b}_{2}{O}_{5}\to Nb{O}_{2}\to NbO\to NbC)$ leads to more evolution of gases, which enhances the porosity on the particle surfaces, which is demonstrated in Fig. 7.

### Spectroscopic analysis

Raman spectroscopy was used to analyze the nature of carbon in samples 5C800 and 10C800. Figure S5 shows the Raman spectra with two characteristic bands corresponding to disordered (D- band) and graphitic carbon (G- band) near 1282.1 nm^−1^ and 1589.8 nm^−1^ respectively. Ferrari and Robertson^67^ suggested that the disordered scattering and bond stretching of sp^2^ carbon atoms contribute to the formation of D- and G- band respectively. The position of the G- band near to 1600 nm^−1^ suggests nano-crystalline graphite. The observed higher intensity of the D- band than the G- band in these samples depicts the amorphous nature of carbon as has also been observed from TEM analysis.

**Figure 8:**
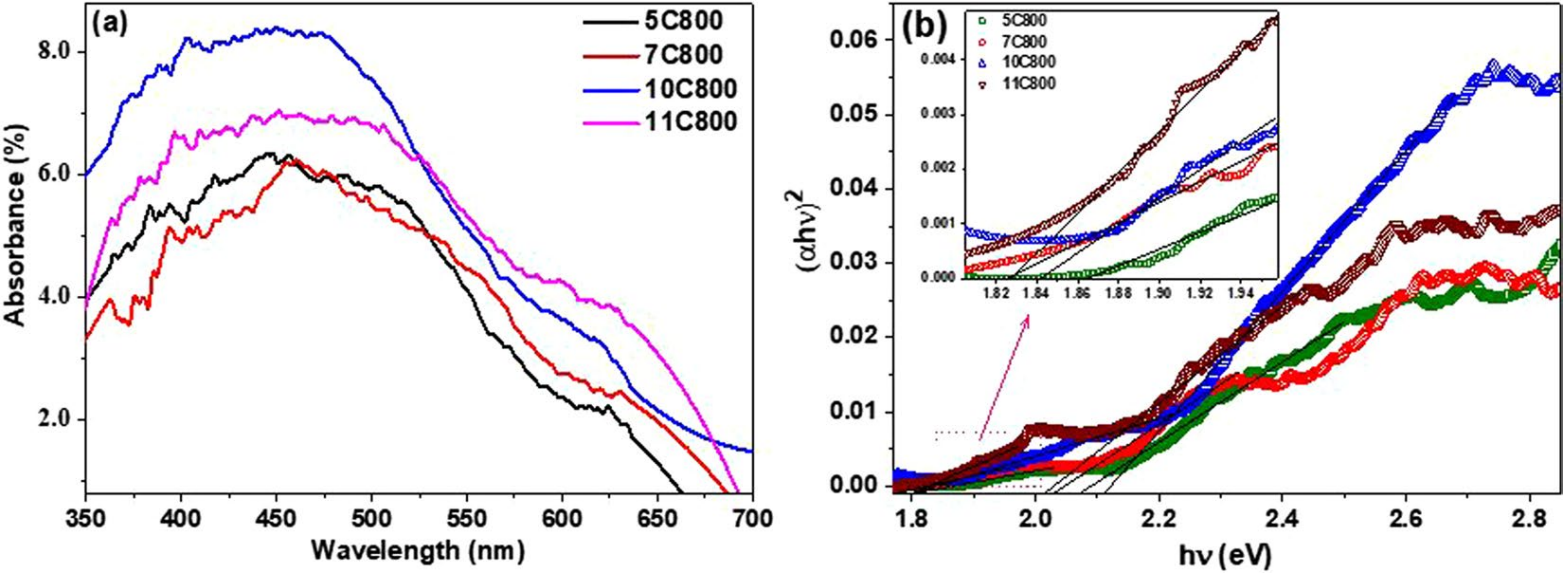
(**a**) UV-Vis absorption spectra and (**b**) Tauc plot depicting dual band-gap corresponding to dual absorbance of all the samples synthesized at 800 °C.

Figure 8a shows the UV-visible absorption spectra of all the samples synthesized at 800 °C suggesting broad multiple absorption humps in the visible region, which may be associated with the presence of either oxide centers (NbO_2_ and Nb_2_O_5_) or continuous carbon network (highly disordered graphic carbon) in the nanocomposite powder samples^68–70^. The band gap for all the samples was calculated with the help of Tauc’s relationship between photon energy (hν) and absorption coefficient (α) as

1 $${\boldsymbol{\alpha }}h{\boldsymbol{\nu }}={\boldsymbol{A}}{(h{\boldsymbol{\nu }}-{{\boldsymbol{E}}}_{{\boldsymbol{g}}})}^{{\boldsymbol{n}}}$$

where A is a constant and ‘n’ depends on the type of transition containing values 0.5, 2.0, 1.5 and 3.0 corresponding to allowed direct, allowed indirect, forbidden indirect, and forbidden direct transitions, respectively^71^.

The values of band gap were calculated by extrapolating the linear portion of the (αhν)^2^ vs. hν curve as shown in Fig. 8b. The absorption observed at 600–650 nm and 550 nm might be associated to the presence of disordered carbon^70^ and Nb-O centers^69^, respectively. Table 4 shows the band gaps (~1.8 eV & ~2.1 eV) of as-synthesized samples depicting the absorption in the visible region of the E-M spectrum, which makes them suitable materials for studying their photocatalytic behavior under visible irradiation.

**Table 4:** Band gap of the samples synthesized at 800 °C.

Sample label	Band gap (eV)	
5C800	1.86	2.09
7C800	1.82	2.05
10C800	1.84	2.12
11C800	1.82	2.02

**Figure 9:**
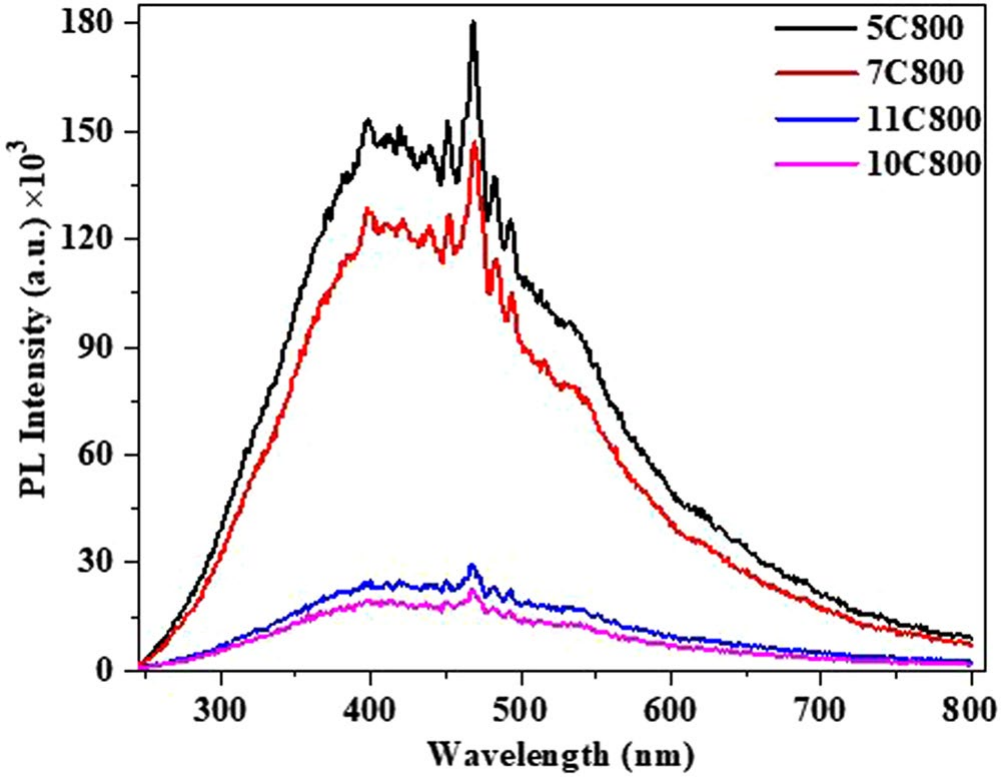
Photoluminescence spectra of all the samples synthesized at 800 °C.

Moreover, to observe the photoemission characteristics of the synthesized samples photoluminescence (PL) spectroscopy was conducted, which is shown in Fig. 9. A broad emission spectrum in the visible region was observed for all the samples, which is associated with disordered carbon (observed in RAMAN and XPS analysis) and other functional groups present in the powder samples. Photoemission decreases as the amount of lattice carbon increases and lattice distortion decreases from a holding time of 5 h (5C800) to 10 h (10C800).

For 11 h of holding time (11C800) more intense photoemission is observed as compared to 10C800. This is because of the presence of some functional groups, which reduce the efficiency of non-radiative decay. The XPS spectra showed the energy corresponding to π → π* transition which also promotes the photoluminescence quenching as observed in Fig. 9 which is in good intact with its volume fraction on the surface of different photo catalysts (5C800 and 7C800)^72^. Moreover, the presence of NbC_x_O_y_ on the surface also promotes the photoluminescence quenching.

### Photocatalytic study

**Figure 10:**
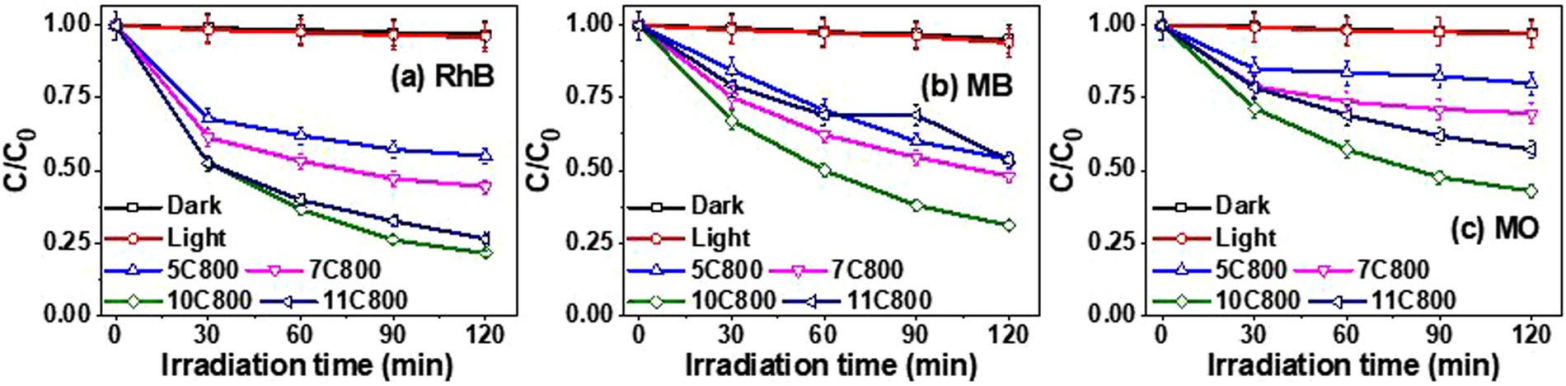
Change in concentration of (**a**) RhB dye, (**b**) MB dye and (**c**) MO dye.

Photocatalytic activity of prepared nanocomposites was monitored by observing the degradation of RhB, MB and MO dyes. All of them are cationic dyes, which acquire electrons from donor excited sites of a photocatalyst. Figure S6 shows the diminishing absorption spectra of dyes (RhB, MB and MO) under the exposure of visible radiation for 120 min which might be associated to the decolorization or degradation of dyes with respect to irradiation time. The fractional variation in the dye concentration after 120 min are shown in Fig. 10(a–c). The controlled experiments showed that ~2.8% decolorization of dyes (without catalyst) was observed in a dark chamber. Moreover, 3.2% photo bleaching was observed under visible irradiation without photocatalyst which is negligible. The experiments under visible irradiation with the as synthesized photo catalysts showed decolorization/degradation as a function of exposure time for the derivatives of RhB, MB, and MO, respectively as shown in Fig. 10(a–c).

**Figure 11:**
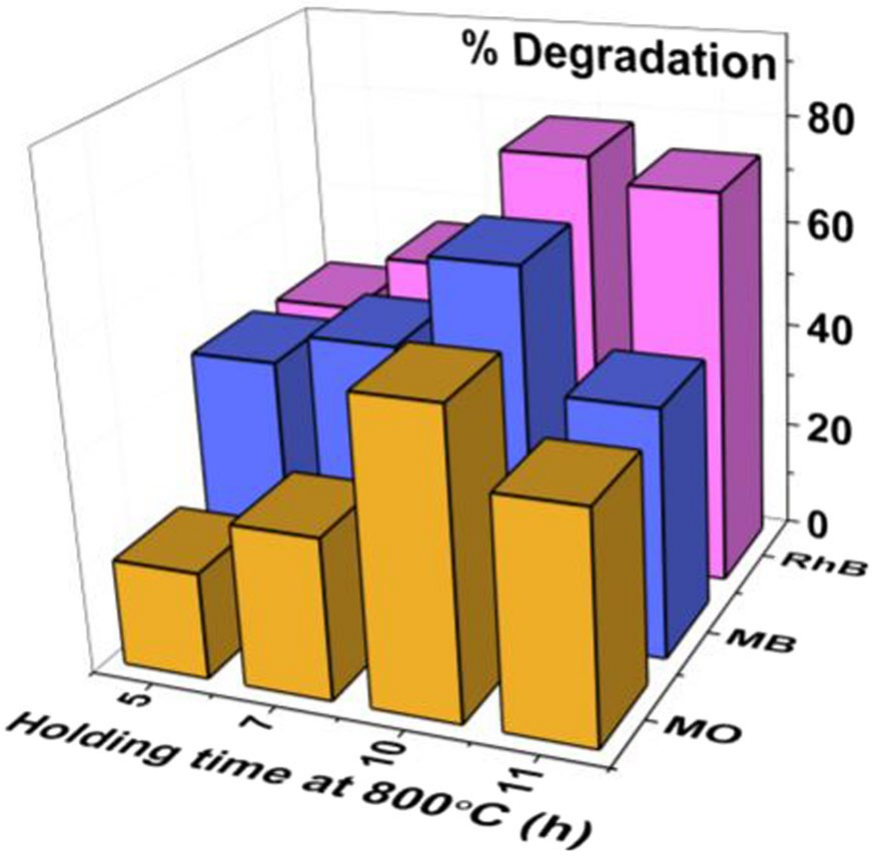
Comparative bar chart of the degradation of dyes after 120 min irradiation.

Moreover, Fig. 11 shows a comparison chart of percent degradation after 120 min of exposure which elucidates the better performance of 10C800 followed by 11C800, 7C800 and 5C800 with 78.6%, 67.8%, and 57.1% change in concentration of RhB, MB, and MO, respectively. The presence of oxides in the samples may affect the generation of excitons and their transfer towards the surface. Sample 5C800 shows the least photocatalytic activity while minor content of oxides in 7C800 and 11C800 resulted in better activity than 5C800. Among all samples, 10C800 shows better photocatalytic activity while other samples contribute differently with all the dyes (Fig. 11). Such behavior may be associated with different molecular structures of the dyes due to generated excitons interacting differently at targeted sites e.g. aromatic rings, N, S and unsaturated bonds etc.

**Figure 12:**
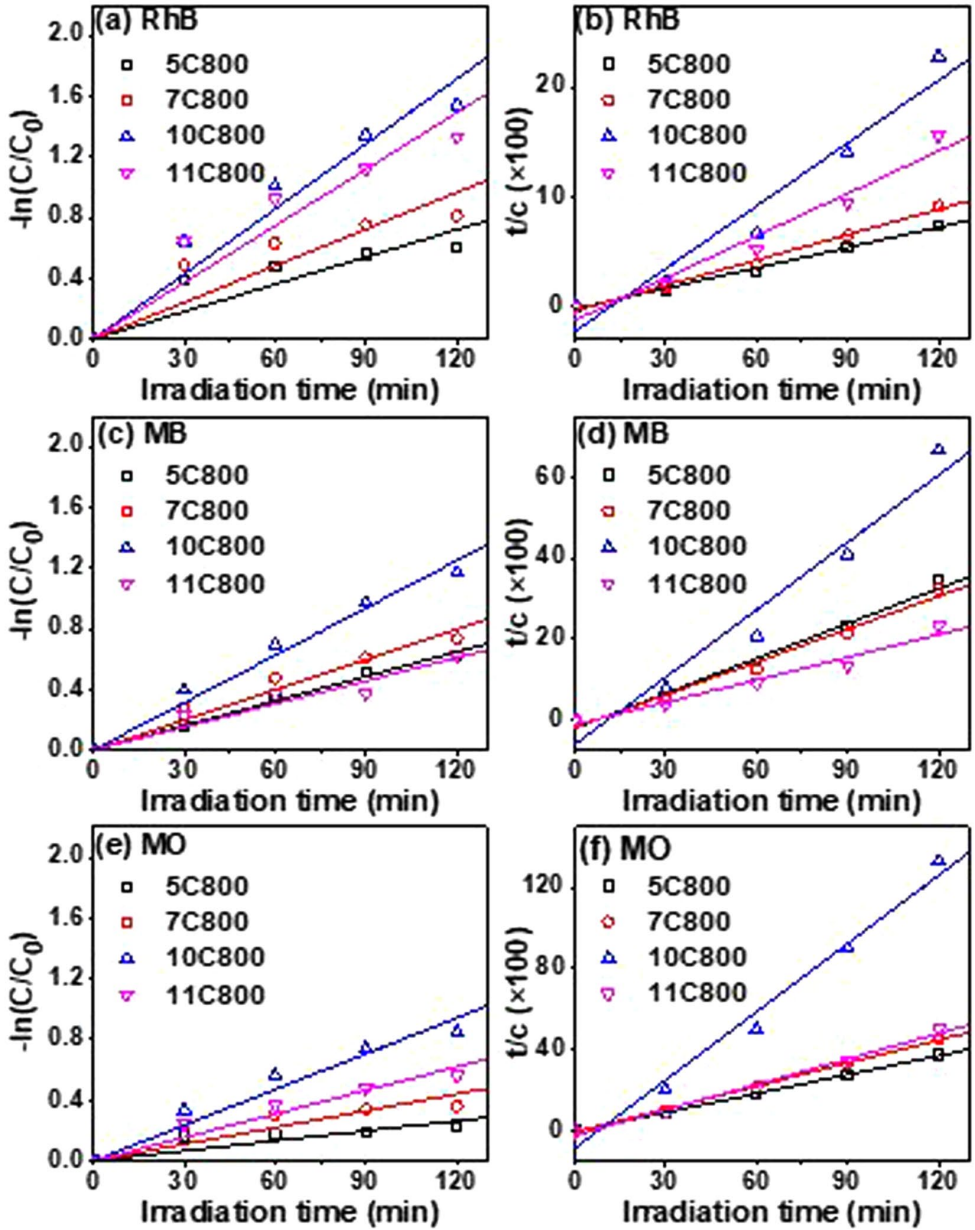
First order kinetics (**a**,**b**,**c**) and second order kinetics (**b**,**d**,**e**) of dyes with all the samples synthesized at 800 °C of (**a**) RhB, (**b**) MB and (**c**) MO.

The decolorization/degradation kinetics of photocatalytic reactions were studied with the help of pseudo first and second order law, which can be expressed as follows:

2 $$-{\bf{In}}(\frac{{\bf{C}}}{{{\bf{C}}}_{{\bf{0}}}})={{\bf{K}}}_{1}{\bf{t}}\,{\rm{and}}\,\frac{{\boldsymbol{t}}}{{\boldsymbol{C}}}=\frac{1}{{{\boldsymbol{K}}}_{{\bf{2}}}{{\boldsymbol{C}}}_{{\bf{0}}}^{{\bf{2}}}}+\frac{{\boldsymbol{t}}}{{{\boldsymbol{C}}}_{{\bf{0}}}}$$

where C_0_, C, K_1_ and K_2_ are the solution concentrations at irradiation time t = 0, at different times and pseudo first and second order rate constant (per minute), respectively^71^. A plot of −ln(C/C_0_) vs. t (min) and t/C vs. t (min) as shown in Fig. 12 represents pseudo first order kinetics (a,c,e) represents the pseudo second order kinetics (b,d,f), respectively. The respective reaction rate constants (K_1_ and K_2_; min^−1^) and regression coefficients (R^2^P) have been listed in Table 5, which illustrated that 10C800 has the highest photochemical reaction rate constants (K_1_) of 0.0143, 0.0102, and 0.0075 min^−1^ for RhB, MB, and MO dyes, respectively, following pseudo first order kinetics. Moreover, based on quality of fitting (R_2_), it can be suggested that 10C800 and 11C800 followed pseudo first order kinetics while, 5C800 and 7C800 followed second order kinetic (Table 5). Similar trend has also been observed by Yang *et al*.^73^ and Younis *et al*.^74^ for photoelectron reduction of Cr^4+^ on MoS_2_@TiO_2_ nanotubes and photodegradation activity of undoped and doped CeO_2_ nanocrystals.

**Table 5:** Pseudo first and second order reactions for degradation of RhB, MB and MO.

	K_1_ (min^−1^) for RhB	R^2^	K_1_ (min^−1^) for MB	R^2^	K_1_ (min^−1^) for MO	R^2^
**Pseudo first order**						
5C800	0.006	0.91	0.005	0.99	0.002	0.88
7C800	0.008	0.92	0.006	0.98	0.003	0.90
10C800	0.014	0.98	0.010	0.99	0.007	0.97
11C800	0.012	0.96	0.005	0.97	0.005	0.97
	**K**_**2**_ **(min** ^**−1**^ **) for RhB**	**R** ^**2**^	**K**_**2**_ **(min** ^**−1**^ **) for MB**	**R** ^**2**^	**K**_**2**_ **(min** ^**−1**^ **) for MO**	**R** ^**2**^
**Pseudo second order**						
5C800	0.219	0.99	0.071	0.97	1.525	0.99
7C800	0.122	0.98	0.078	0.97	2.120	0.99
10C800	0.021	0.93	0.026	0.94	0.152	0.97
11C800	0.038	0.94	0.119	0.95	0.394	0.98

**Figure 13:**
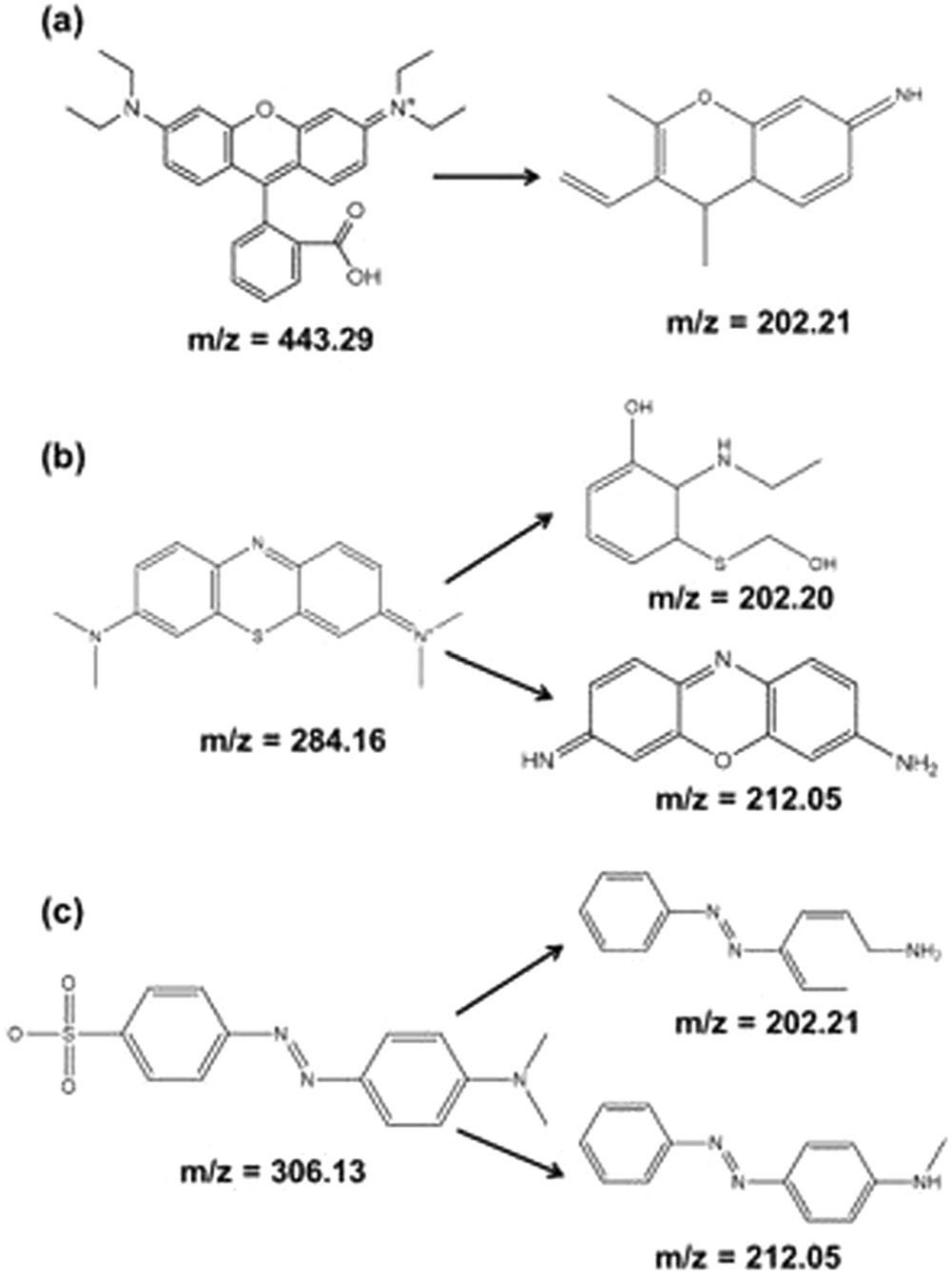
Probable degraded products for (**a**) RhB, (**b**) MB and (**c**) MO after exposer of 120 min.

**Figure 14:**
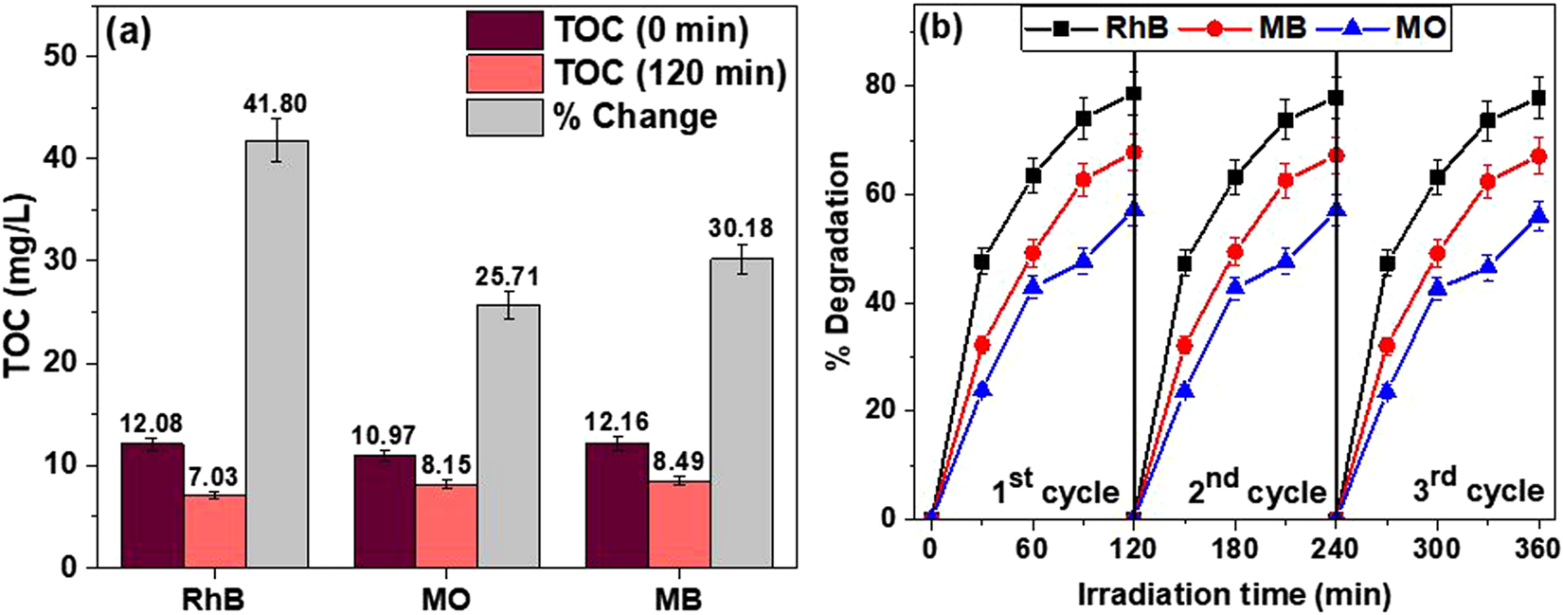
(**a**) Total organic carbon (TOC) of different dyes with 10C800 before and after the 120 min visible irradiation; (**b**) Reusability of 10C800 as photocatalyst under visible irradiation.

Moreover, the observed diminishing absorption patterns may be associated with the interaction of excitons with different reactive oxygen species (ROS) such as hydroxyl radicals (OH^·^) and superoxide anion radicals (${O}_{2}^{\cdot -}$). With the help of mass spectrometry, the decreasing absorption spectra were attributed to the degradation of dye molecules to lighter organic molecules which are shown in Fig. 13 as lower m/z molecules along with the parent molecule (Figs S7–S9). To ascertain the results obtained from absorption spectra and MS analysis, TOC was carried out to check the decrease of concentration of organic carbon. In association with the MS results, 41.8%, 25.71%, and 30.18% reductions were observed for concentrations of organic carbon irradiation in RhB, MO, and MB dye solution, respectively, after 120 min as shown in Fig. 14a. These results demonstrate the partial degradation of parent dye molecules due to the presence of some organic carbon in the form of aromatic rings which can be asserted that the decolorization (absorption study) rate was faster than the degradation (MS and TOC study) rate of dye molecules.

The photocatalytic activity of the synthesized samples was increased with increasing holding time at 800 °C up to 10 h, which again was decreased at 11 h. As observed from XRD analysis, carbon content in the lattice of NbC has increased from 0.79 (5C800) to 0.959 (10C800) resulting in less distorted crystallites (Table 2) which is further decreased at longer holding time (11 h) with higher distortion as discussed in the W-H analysis. Moreover, a similar trend was also observed from BET analysis where SSA was increased from 292 m^2^/g (5C800) to 506 m^2^/g (10C800) and then decreased to 475 m^2^/g (11C800). Further, PL spectroscopy data show that recombination decreases with the decreased impurities in the synthesized samples resulting in better mineralization of dye with 10C800 than other samples. Further, the reusability of the nanocomposite photocatalyst is a significant parameter which maneuvers the practical usefulness of photocatalyst. To observe the reusability and stability of as-prepared C coated NbC NPs, recycle reactions were carried out for the degradation of all the dyes over 10C800 as shown in Fig. 14b. To observe the recyclability of as prepared photocatalyst, previously used photo catalysts (10C800) were removed from the solution by centrifugation and then reused for the photodegradation of all the dyes with the same concentration of the solution and irradiation intensity. The excellent stability of photocatalyst (10C800) is well-illustrated from the XRD pattern of 10C800 after the 3^rd^ cycle of photodegradation reaction as shown in Fig. S10. These results suggest sufficient stability of the photocatalyst during photodegradation of the dyes and can be reused without significant decrease in performance.

**Figure 15:**
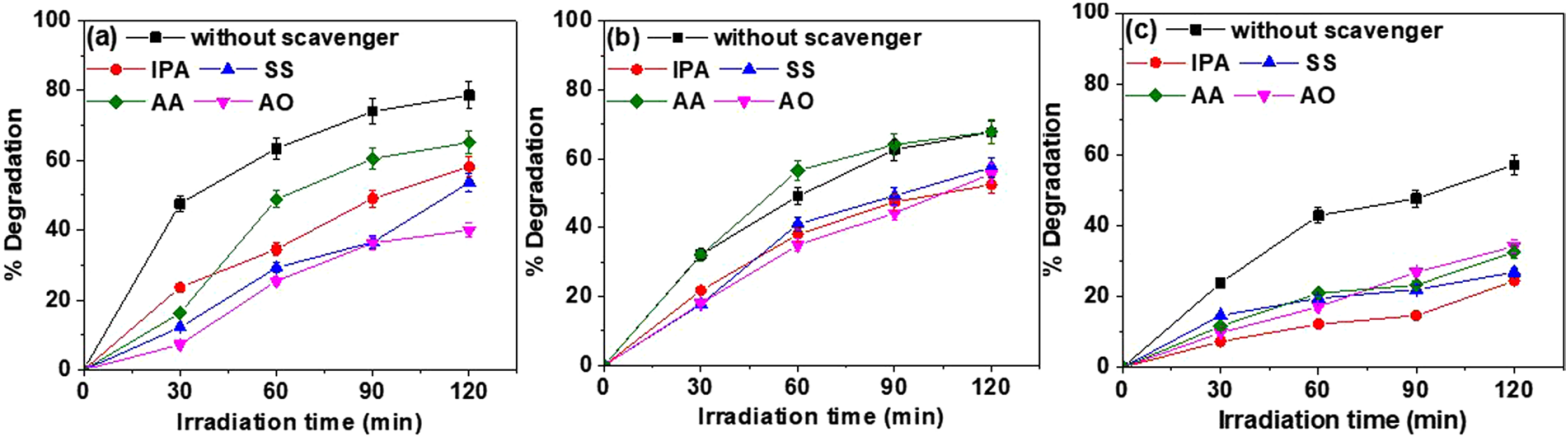
Effect of various scavengers on the degradation of (**a**) RhB, (**b**) MB and (**c**) MO dye with photocatalyst (10C800).

To establish the mechanism responsible for such a good photocatalytic behavior of synthesized photocatalyst, the generation of reactive oxygen species (ROS) has to be confirmed by using e^−^, h^+^, OH^·^ and ${O}_{2}^{\cdot -}$ scavengers. As an effect of these scavengers, degradation of dyes got retarded differently, which is shown in Fig. 15. In case of RhB dye, the addition of AO (hole scavenger) in dye solution retarded the degradation rate by 38% as compared to other scavengers and degradation of MB dye was reduced by 15% with the addition of SS, IPA, and AO (Fig. 15a,b, respectively). Furthermore, addition of superoxide free radical scavenger i.e. AA does not affect the degradation of MB dye. On the other hand, degradation of MO dye was affected significantly in the presence of scavengers among which IPA reduced the efficiency by 33% which is higher than other scavengers (Fig. 15c). The observed retardation in photocatalytic degradation of different dyes can be correlated with the generation of the above-mentioned ROS.

### Degradation mechanism

**Figure 16:**
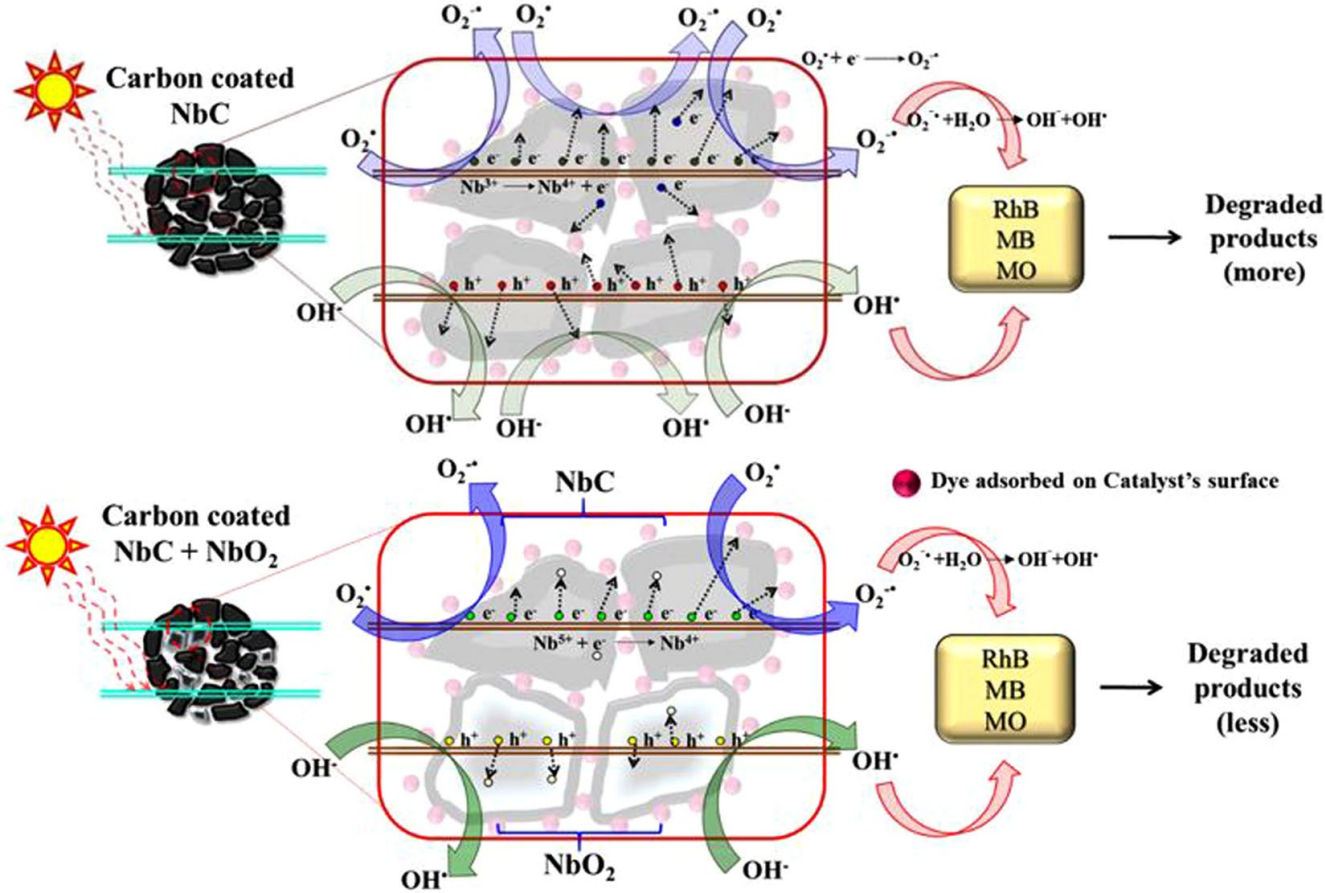
Mechanism of the mineralization of dyes (RhB, MB and MO).

Figure 16 illustrates the proposed mechanism responsible for the photocatalytic degradation of dyes under visible light irradiation. As per the XRD results, it cannot be stated that nanocomposite sample consists of NbC alone, but, presence of O-centers (observed form TEM and XPS analysis) inside the NPs induced the optical active sites generating the charge carriers upon visible exposure. Earlier, Ohgi *et al*.^75^ and Ishihara *et al*.^76^ also suggested the enhanced generation and transportation of charge carriers by inducing oxygen centers (through partial oxidation) in the carbide nanoparticles. Figure S11 shows the valence band XPS spectra of 10C800 and 5C800 depicting different VB edges of ~1.97 and ~1.71 eV and with the help of optical absorption analysis, the position of the CB can be estimated. The CB edge potential of both the samples (−0.15 eV and −0.38 vs NHE, respectively) are more negative than the standard redox potential of ${{\rm{O}}}_{2}/{{\rm{O}}}_{2}^{-}$ (−0.046 eV vs NHE) to reduce molecular oxygen^77^. Further, VB potential edge of ${{\rm{O}}{\rm{H}}}^{-}/{}^{.}{\rm{O}}{\rm{H}}$ (+1.99 eV vs NHE) is lower than VB of photocatalyst^77^. Being metallic in nature, NbC supported the charge transfer towards the surface of NPs where the reaction with the hydroxyl anion and dissolved oxygen in water produces hydroxyl free radicals (${{\rm{OH}}}^{\cdot }$) and superoxide free radical anions (${O}_{2}^{.-}$), respectively. These reagents are considered as strong oxidizing agents and thus oxidize the dyes adsorbed on the surface of the NPs. Various reactions, which are responsible for the degradation of dyes and are expressed as follows:

3 $${\boldsymbol{Nb}}{\bf{C}}+{\boldsymbol{C}}+{\bf{h}}{\boldsymbol{\nu }}\to {\bf{N}}{\bf{b}}{\bf{C}}+{\bf{C}}+{{\bf{h}}}^{+}+{{\bf{e}}}^{-}$$

4 $${{\bf{O}}}_{2}^{\cdot }+{{\bf{e}}}^{-}\to {{\bf{O}}}_{2}^{\cdot -}$$

5 $${{\bf{O}}}_{2}^{\cdot -}+{{\bf{H}}}_{2}{\bf{O}}\to {\bf{O}}{{\bf{H}}}^{\cdot }+{\bf{O}}{{\bf{H}}}^{-}$$

6 $${\bf{O}}{{\bf{H}}}^{-}+{{\bf{h}}}^{+}\to {\bf{O}}{{\bf{H}}}^{\cdot }$$

For all the samples, degradation efficiency is a function of phase content (NbC, NbC_x_O_y_, and Nb-O), π→π* transitions and oxygen vacancies present on the surface. Among all of these parameters, the presence of NbC_x_O_y_ and π→π* governs whole catalytic reaction (as discussed in PL analysis). Moreover, oxygen vacancies promotes the delayed recombination (PL emission peak at 468 nm) which in turn provide more excitons^78^. But, excess vacancies can also quench the photocatalytic reaction which might be responsible for the observed reduced photodegradation in 7C800 as compared to 10C800. While, 11C800 possess insufficient vacancies providing lesser degradation efficiency than 10C800.

Except for 10C800, both 7C800 and 11C800 contain higher carbonyl (C=O) group, considered as hole scavengers^79,80^, which in turn reduce the generation of OH^·^. Since, OH^·^ is a strong oxidizing agent as compared to ${{\rm{O}}}_{2}^{\cdot -}$, reduction in the generation of OH^·^ retards the photocatalytic degradation efficiency of 7C800 and 11C800. Furthermore, in 7C800 and 11C800, Nb exhibits 4+ (NbC_x_O_y_, NbC, NbO_2_) and 5+ (Nb_2_O_5_) oxidation states in which Nb^5+^ tries to attain a more stable state, i.e. Nb^4+^ by scavenging an electron to retard the photodegradation. Sample 10C800 consists of Nb in the 3+ (NbC_x_O_y_, NbC) and 4+ (NbC_x_O_y_, NbC, NbO_2_) states in which Nb^3+^ will try to achieve Nb^4+^ by ejecting an electron to enhance the degradation efficiency which can be expressed by the following equations:

7 $${\bf{N}}{{\bf{b}}}^{5+}+{{\bf{e}}}^{-}\to {\bf{N}}{{\bf{b}}}^{4+}$$

8 $${\bf{N}}{{\bf{b}}}^{3+}\to {\bf{N}}{{\bf{b}}}^{4+}+{{\bf{e}}}^{-}$$

As the degradation can be achieved by the transformation of chromophore groups and the generation of energetic electrons in visible light, photocatalysis is thermodynamically and kinetically limited which might cause the partial degradation of RhB, MB and MO dyes even with the visible range band gap of photo catalysts based on MS and TOC results^81^. With the help of results of detection of ROS species, the generation of ROS species is a function of both photocatalyst and dye solution. In the case of MO dye, all the species are being generated among which hydroxyl radical is the most active oxidizing agent. But, degradation of MB dye occurred by the hole, electron, and hydroxyl radical equivalently where, holes dominated the degradation of RhB dye as compared to other ROS species. Thus, smaller crystallite size, high pore volume and specific surface area, smaller recombination, and fine tuning of bandgaps are considered to be prominent factors for the enhanced photocatalytic properties of single phase carbon coated NbC NPs. Further, to check the toxicity of the best degraded dye solutions where 10C800 photocatalyst was used, a toxicity test using IS: 6582-2001 method was done. In the adopted method, 100% survival of fish species was observed after 96 h indicating the non-toxicity of the degraded solution as shown in Fig. S12.

## Summary

From a series of experiments, carbon coated NbC_x_ NPs has been successfully synthesized by using Nb_2_O_5_, Mg, and charcoal at relatively low temperature (800 °C). However, the holding time at 800 °C for the synthesis has been optimized as 10 h to obtain carbon coated NbC_x_ NPs in which x = 0.95 is obtained. The distorted lattice due to simultaneous transportation of carbon and oxygen has been supported by W-H analyses having the least distortion in 10C800. The as synthesized NPs are thermally stable up to 400 °C in air atmosphere (Fig. S4). The existence of multiple phases in XRD patterns at different temperatures and holding times conveys a multistep *in situ* reduction-carburization process for the formation of NbC. This results in agglomerated mesoporous NPs. The XRD of 10C800 sample shows the formation of single phase NbC, however, XPS results show the multiple oxidation states of Nb (associated to NbC, NbC_x_O_y_, NbO_2_ and Nb_2_O_5_) along with the both sp^2^ and sp^3^ hybridized carbon, oxygen vacancies confirming the removal of oxygen from Nb_2_O_5_ (reactant). Raman spectroscopy also confirmed the presence of disordered graphitic carbon as coatings of NPs. UV-visible spectroscopy affirmed the absorption of visible radiation corresponding to small bandgaps. The higher O-vacancies, less π → π* transition and lower NbC/NbC_x_O_y_ content are responsible for the increased PL emission intensity in 5C800, 7C800, and 11C800 as compared to 10C800. Small crystallites, high SSA, and less PL emission intensity gave enhanced photocatalytic properties of optimized sample (10C800) that follow pseudo first order kinetics. Further, visible light photocatalysis is very complex phenomenon comprising of decolorization and degradation of dye solution with the help of photocatalyst. For visible light photocatalysis with synthesized single phase NbC (10C800), rate of decolorization is faster than that of degradation of dye solution in 120 min under visible irradiation, which is much better than that reported by Chen *et al*.^52^.

## Experimental Methods

### Synthesis

To synthesize carbon coated NbC nano-composite, Nb_2_O_5_ (1.329 g), metallic Mg powder (2.0 g) and activated charcoal (1.5 g) were used as the niobium source, reducing agent, and carbon source respectively. The mixture of reactants was heated to different temperatures (600, 700 and 800 °C) at a heating rate of 5 °C/min for different holding times (5, 7, 10 and 11 h) in a specially designed stainless steel autoclave^50^. The autoclave was allowed to cool within the furnace to room temperature. Thereafter, the obtained black powder was leached in 50% diluted HCl (v/v) and then washed with distilled water. The washed powder sample was further dried at 120 °C. The obtained powder samples were assigned as 10C600, 10C700, 5C800, 7C800, 10C800, and 11C800 corresponding to their synthesis temperature and holding time as listed in Table 1.

### Characterization

For the identification of phases, X ray diffraction (XRD) analysis of synthesized samples was performed on a PANalytical Xpert-Pro diffractometer with Cu-Kα radiation (λ = 1.5406 Å, Ni filter, step size = 0.1301°) over 20 ≤ θ ≤ 80° and X’pert High Score was used to match the diffraction peaks with standard ICDD cards. Surface chemical compositions of synthesized samples (synthesized at 800 °C) were analyzed by X-ray photoelectron spectroscopy (XPS) (PHI 5000 Versa Prob II, FEI Inc.) using Al-Kα radiation (1486.7 eV) and C1s (284.5 eV) for the calibration of binding energies of all the elements. All the high resolution XPS spectra were analyzed with the help of XPSPEAK41 software by considering the equal FWHM of doublet peaks of Nb3d keeping area ratio of 3:2 (d_5/2_:d_3/2_) with doublet separation of 2.74 eV. Morphologies of synthesized powder samples were observed with transmission electron microscopy (TEM) (JEOL 2100 F, operating at 200 kV). Line profile of nanoparticle was also taken using Cu grid in STEM mode to study the linear distribution of elemental concentration. Thermal analysis (TG) of the samples were carried out with a NETZSCH STA 449F3 at a heating rate of 5 °C/min up to 850 °C in air atmosphere to predict the thermal stability as a function of temperature. The surface area and pore size distribution calculations were done with the help of N_2_ adsorption-desorption analyses (Tristar 3000). Optical absorption spectra of the samples were recorded on a double beam UV-Visible spectrophotometer (Hitachi U-3900H) in the range of 350–700 nm. Raman spectroscopic signals were recorded with a Renishaw Raman Microscope at 50X magnification with 785 nm (wavelength) laser source over a range of 100–1800 cm^−1^.

### Photo-catalysis

For photocatalytic studies, a concentration of 1 mg/L of dye (RhB, MO and MB) was used. The photocatalyst (0.002 g) was suspended in the 100 mL aliquot after homogenizing the dye solution. For the establishment of adsorption-desorption equilibria, the suspended solution was stirred for 60 min in a dark chamber. Thereafter, the solution was exposed to visible radiation of around 8900 lux intensity for 120 min in which the sample aliquots were extracted at an interval of 30 min. The photo reactor was open to air atmosphere to avoid the depletion of dissolved oxygen during the irradiation. Each sample was centrifuged at 4500 rpm to extract photocatalyst from the suspension and supernatant was characterized with a double beam UV-Visible spectrophotometer (Hitachi U-3900H) to observe the absorption spectra of dye solution at regular time interval. To confirm the variation of absorbance of dye solution due to decolorization or degradation, the filtered aliquots (WHATMAN 0.45 µm, PTFE) after 120 min irradiation were characterized by mass spectroscopy (MS) and total organic carbon (TOC). MS was carried out on UPLC-XEVO-G2-XS/QTOF mass spectrometer equipped with electron ionization source operated in positive ion mode. The mass spectrometer ion source and desolvation temperatures were set to 120 °C and 350 °C, respectively. Further, TOC was measured by MULTI N/C 3100 (N3-800/O) with the gas flow rate 160 mL/min and furnace temperature 800 °C. Further, the generation of reactive oxidative species (ROS) in the photodegradation of dyes were observed with *in situ* trapping experiments. The detection process was similar to the photodegradation experiments in which four different scavengers, such as, isopropanol (IPA, hydroxyl free radical scavenger, 1 mM), sodium sulphate (SS, electron scavenger, 1 mM), ammonium oxalate (AO, hole scavenger, 1 mM) and ascorbic acid (AA, superoxide radical scavenger, 1 mM) were added to dye solution in a 1:10 volume ratio prior to irradiation in separate systems.

## Electronic supplementary material

Supplementary Information
