# Supplementary material for: Low temperature synthesis of NbC/C nano-composites as visible light photoactive catalyst
Source: Sci Rep. 2018 Sep 11;8:13597. doi: 10.1038/s41598-018-31989-z (PMC6133931; doi:10.1038/s41598-018-31989-z)
Supplement: Supplementary file 1 — Supplementary Information [file 41598_2018_31989_MOESM1_ESM.docx]

**Low temperature synthesis of NbC/C nano-composites as visible light photoactive catalyst**

Aayush Gupta^a^, Manish Mittal^a^, Mahesh Kumar Singh^b^, Steven L. Suib^c^ and Om Prakash Pandey^a,*^

*^a^School of Physics and Materials Science, Thapar Institute of Engineering and Technology, Patiala-147004.*

*^b^Department of Mechanical Engineering, Indian Institute of Science, Bangalore-560012.*

*^c^Department of Chemistry, University of Connecticut, 55 North Eagleville Rd., Storrs, Connecticut 06269.*

*Corresponding author:

O.P.P.: oppandey@thapar.edu

**Supplementary Information**

**Figure S-1:** Survey spectra of (a) 5C800, (b) 7C800, (c) 11C800; HR-XPS spectra of Nb3d for (d) 5C800, (e) 7C800, (f) 11C800; HR-XPS spectra of C1s for (g) 5C800, (h) 7C800, (i) 11C800;.and HR-XPS spectra of O1s for (j) 5C800, (k) 7C800, (l) 11C800. To calculate the volume fraction of particular functional group on in HR-XPS spectra following mathematical formula has been used (which has also been incorporated in supplementary information);

| **Nb3d HR-XPS spectra** | $\%Volume fraction of NbC in 10C800= \frac{area of peaks corresponding to NbC ({3d}_{3/2}+{3d}_{1/2})}{total area of peaks observed in Nb3d spectra}\times100$ |
| --- | --- |
| **C1s or O1s HR-XPS spectra** | $\%Volume fraction of C {sp}^{2} in 10C800= \frac{area of C {sp}^{2} peak}{total area of peaks observed in C1s spectra}\times100$ |

3















**Figure S-2:** General scans of 10C800 showing the pressence of NbC at core and amorphous carbon as coating of NbC. Highlighted area in last TEM micrograph is consider for the elemental analysis which has been discussed in main manuscript (section 3.4).

**
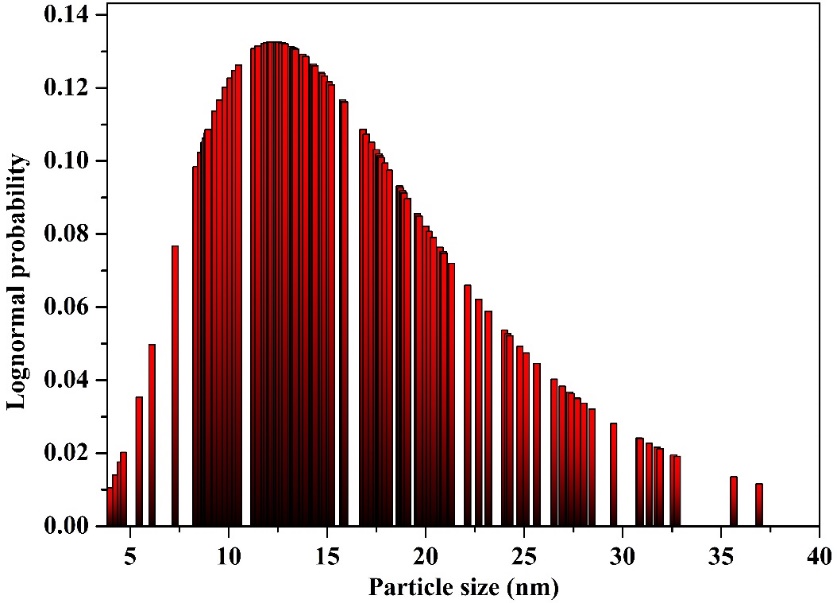
**

**Figure S-3**: log normal distribution of particles of carbon coated NbC nanoparticles.

The median particle size as measured with the help of AxioVision Rel. 4.9.1.0 was 12 nm where a narrow particle size distribution is observed from TEM micrographs, so representing this is complicated. **Figure** **S-3** shows the frequency variation of particle size with the logarithmic size of the particles. The particle size follows the log normal distribution function [2];

| $\boldsymbol{f(d) =}\frac{\boldsymbol{1}}{\sqrt{\boldsymbol{2}\boldsymbol{\pi}}\boldsymbol{.\sigma.}\boldsymbol{d}_{\boldsymbol{i}}}\boldsymbol{e}^{\boldsymbol{-}\left( \frac{\left( \log\boldsymbol{(}\boldsymbol{d}_{\boldsymbol{i}}\boldsymbol{)}\boldsymbol{-\mu} \right)^{\boldsymbol{2}}}{\boldsymbol{2}\boldsymbol{\sigma}^{\boldsymbol{2}}} \right)}$ | (i) |
| --- | --- |
| where $\boldsymbol{\mu=}\frac{\sum\boldsymbol{log(d}_{\boldsymbol{i}}\boldsymbol{)}}{\sum\boldsymbol{n}_{\boldsymbol{i}}}$**; and** $\boldsymbol{\sigma=}\sqrt{\frac{\sum\left( \log\left( \boldsymbol{d}_{\boldsymbol{i}} \right)\boldsymbol{-\mu} \right)^{\boldsymbol{2}}}{\sum\boldsymbol{n}_{\boldsymbol{i}}}}$ | (ii) |

f(d) = log normal distribution of particle size, d_i_ = size of i^th^ particle of NbC, ∑n_i_ = total number of particles under consideration, μ = mean diameter and σ = standard deviation of particle size. Due to very high agglomeration, different criteria provide different average particle sizes.

# **Thermal analysis**

**

**

**Figure S-4:** TG analysis of all the samples.

Thermal behavior of as synthesized samples with variation in temperature and holding time is shown in **Fig. S-4**. The TG/DSC analysis was carried out in air atmosphere with the heating rate of 5 °C/min. All the samples possess some adsorbed water and volatile surface contaminations which were emitted up to 300 °C showing mass loss to some extent (3-7%) and beyond 300 °C, mass gain was observed. Due to the presence of more distortion, sample 10C700 tends to adsorb oxygen while 10C800 assist the delay in oxygen adsorption. Further, sudden mass loss beyond 450 °C corresponds to the simultaneous carbon loss and oxidation of NbC [1]. Higher mass loss of 46% was observed for 10C700 sample synthesized at 700 °C due to more free carbon content as compared to 10C800 (38%) synthesized at higher temperature (800 °C) having pure NbC phase.

Moreover, as a function of holding time at 800 °C, mass gain and loss for all the samples have similar trends except their corresponding temperature range associated to the respective constituents. Among all the configurations opted to observe the effect of holding time, 5C800 showed the steepest and the highest mass loss of 45.7% (**figure S-4**) due to the presence of distorted NbC, NbO_2_ and free carbon. While, an increment in holding time i.e. 7C800 contained less amount of NbO_2_ resulting in less mass gain and mass loss. Due to the carburization of retained NbO_2_ inside the particle at higher holding time, the porous surface of particles may result in a larger extent of oxygen adsorption showing higher mass gain followed by mass loss which may be linked to the higher NbC content in 10C800. Further enhancement in holding time (sample 11C800) decreased the adsorption of oxygen which may again be associated with the presence of NbO_2_ in this sample.


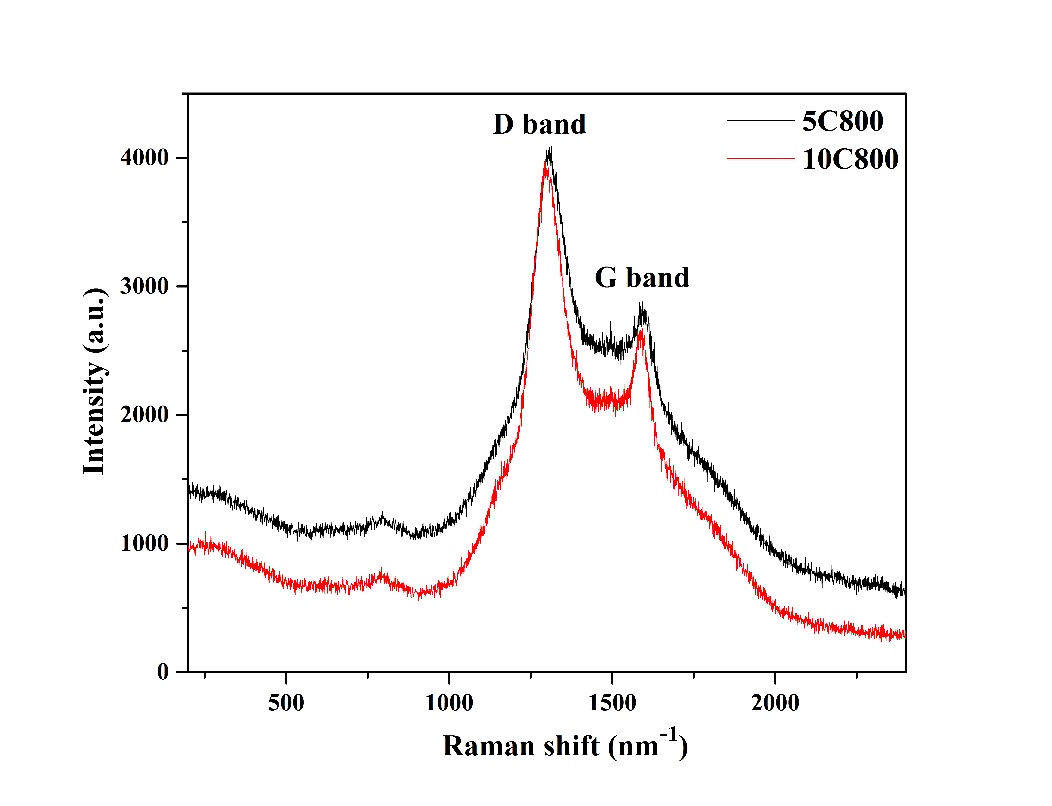


**Figure S-5:** RAMAN spectra depicting the presence of D- and G- band in 5C800 and 10C800.


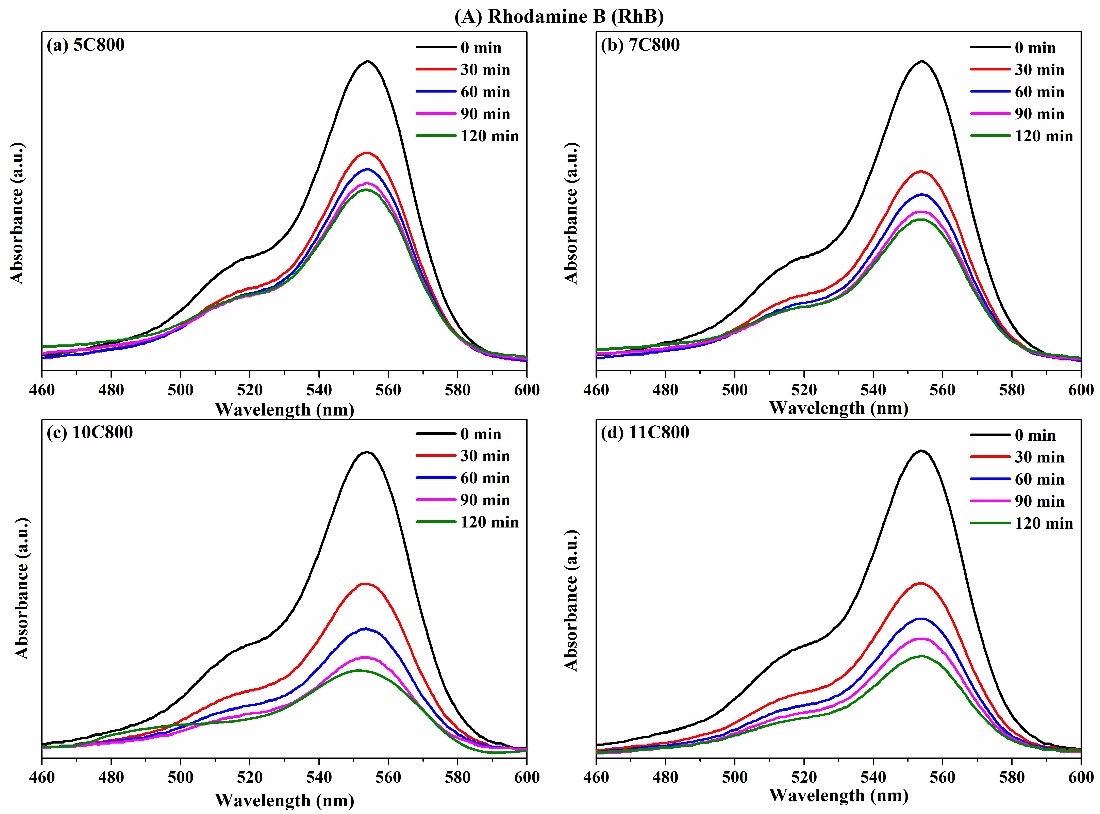


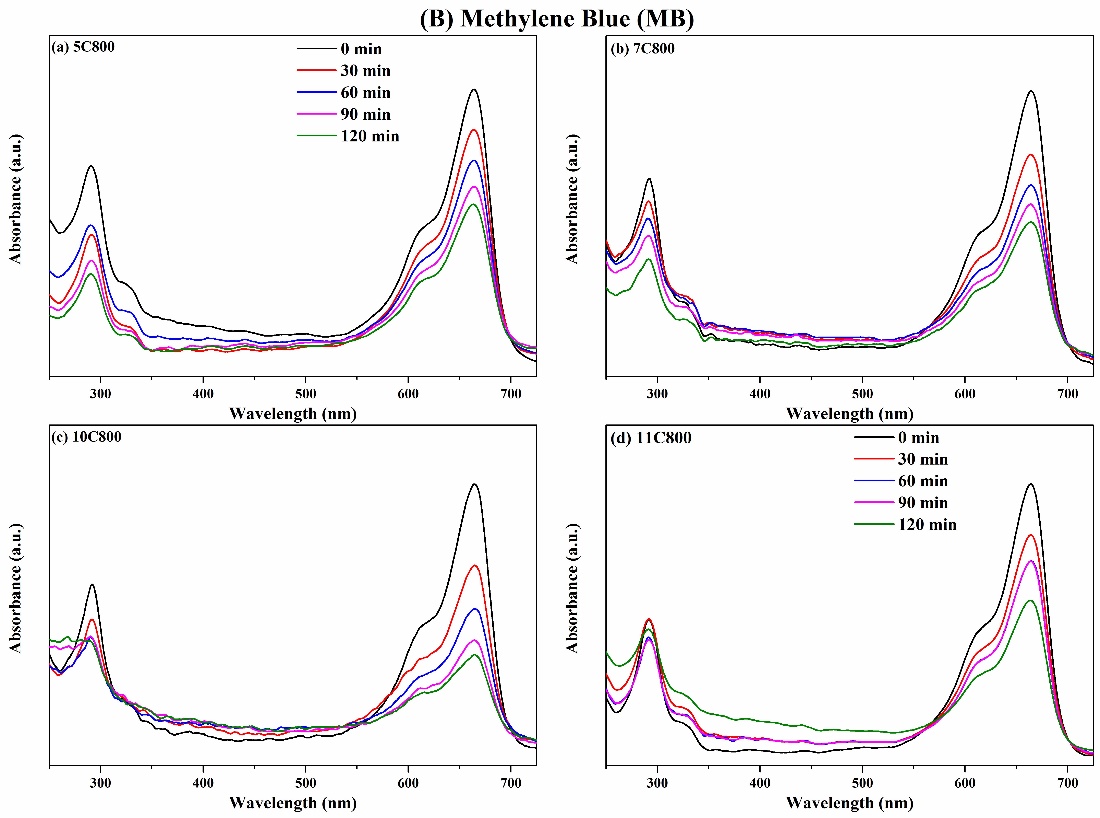


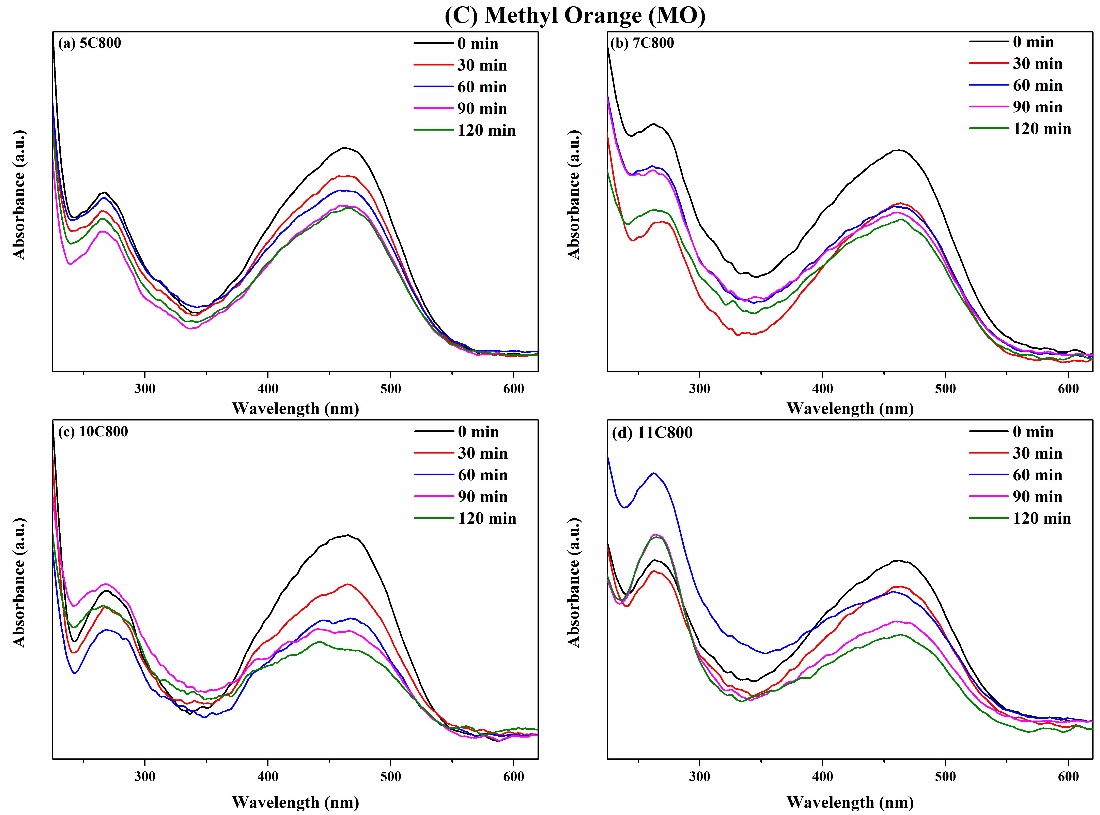


**Figure S-6:** UV-vis absorption spectra showing mineralization of dyes (RhB, MB and MO) in 120 min with various samples (a) 5C800; (b) 7C800; (c) 10C800 and (d) 11C800.


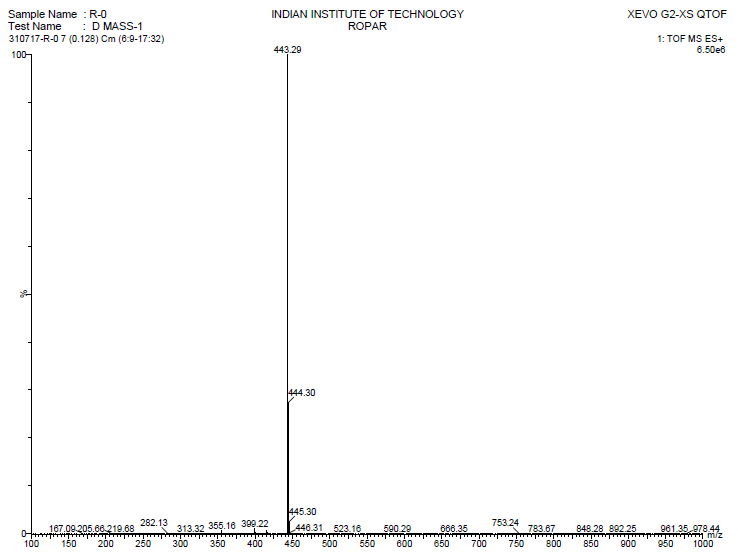


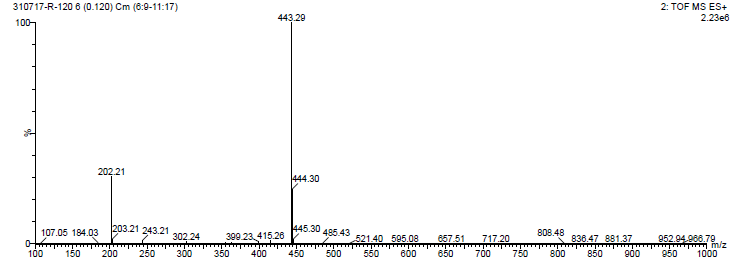


**Figure S-7**: Mass spectrometric results of rhodamine b (RhB) before and after the visible irradiation.


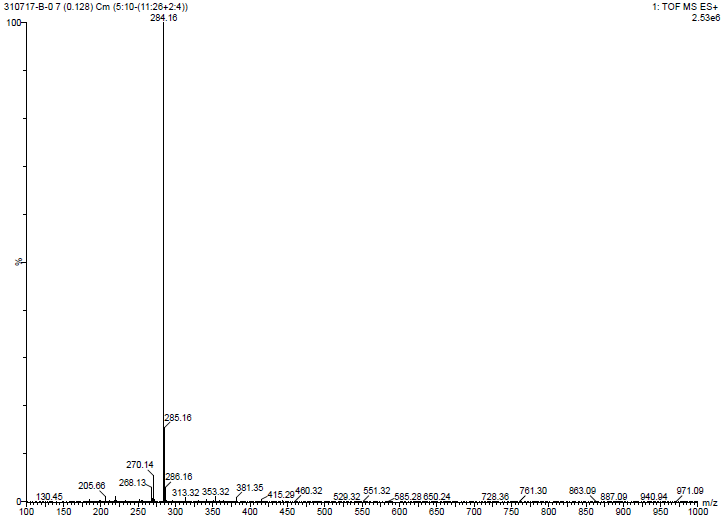


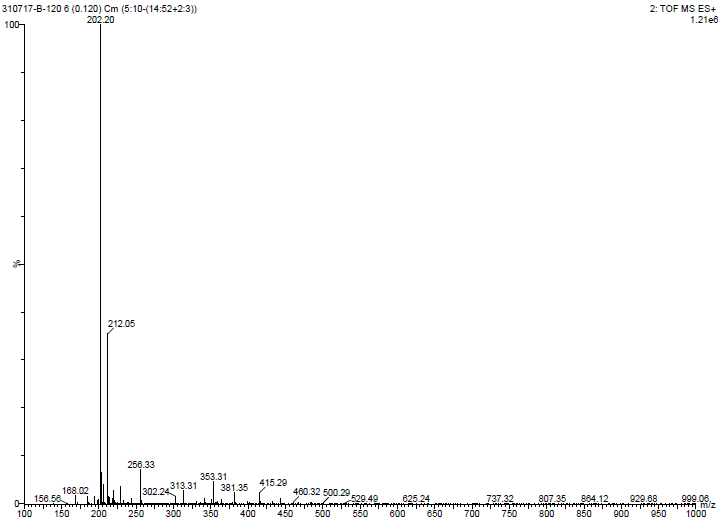


**Figure S-8**: Mass spectrometric results of methylene blue (MB) before and after the visible irradiation.


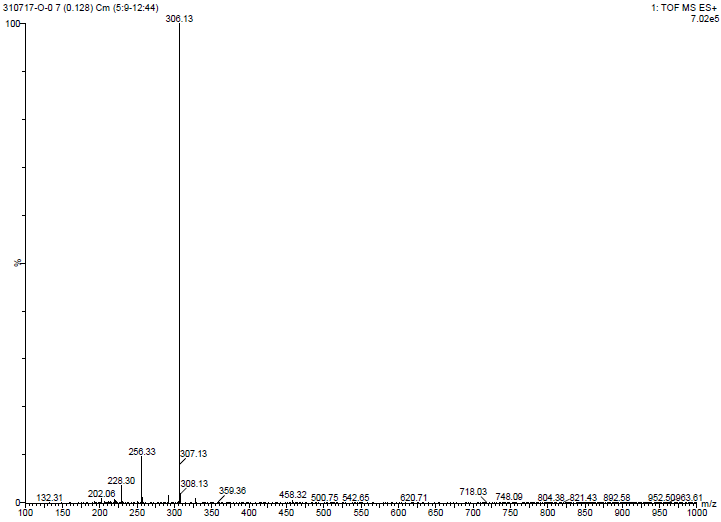


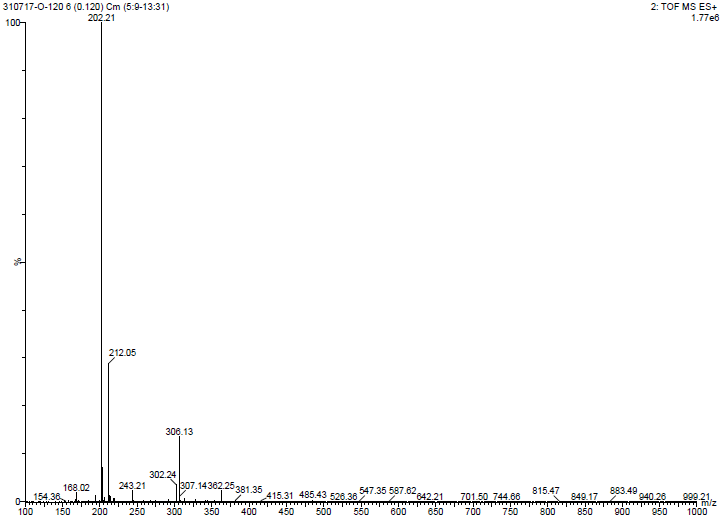


**Figure S-9**: Mass spectrometric results of methyl orange (MO) before and after the visible irradiation.

**Figure S-10:** XRD pattern of 10C800 before and after the photocatalytic reaction.


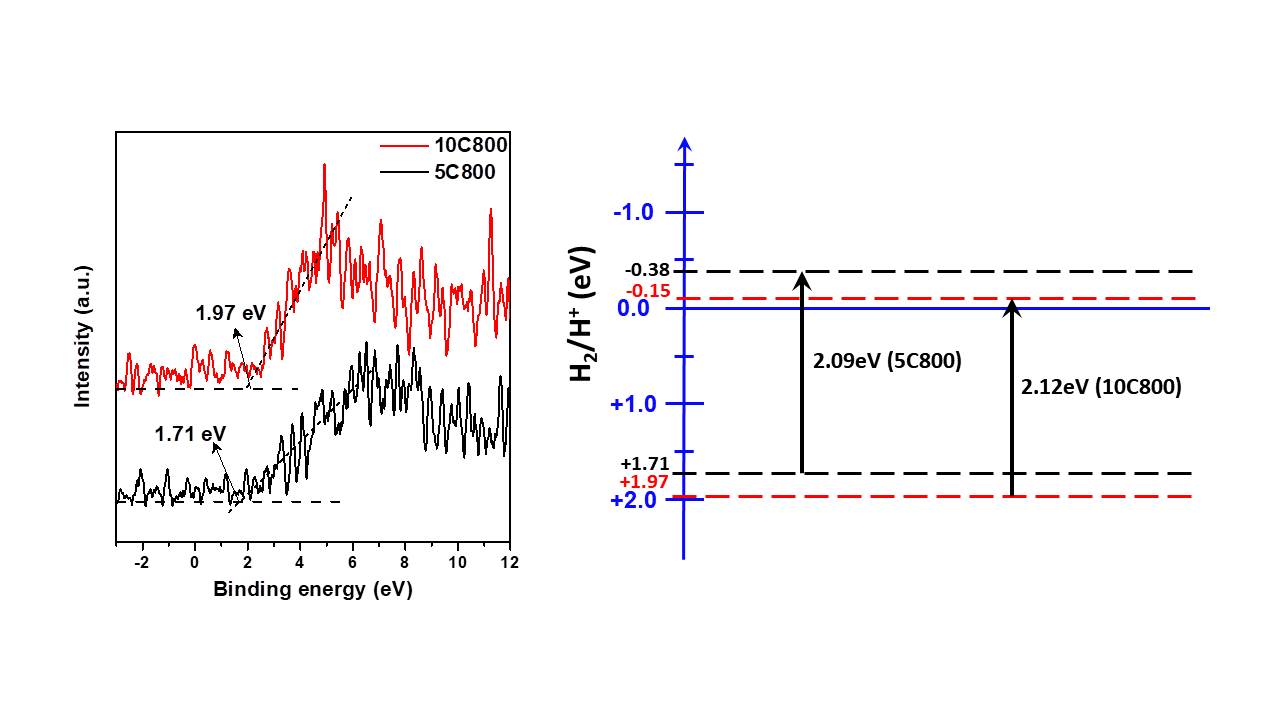


**Figure S-11:** Valence band spectra of 5C800 and 10C800 along with the estimated band structure of synthesized nanocomposite as photocatalyst.


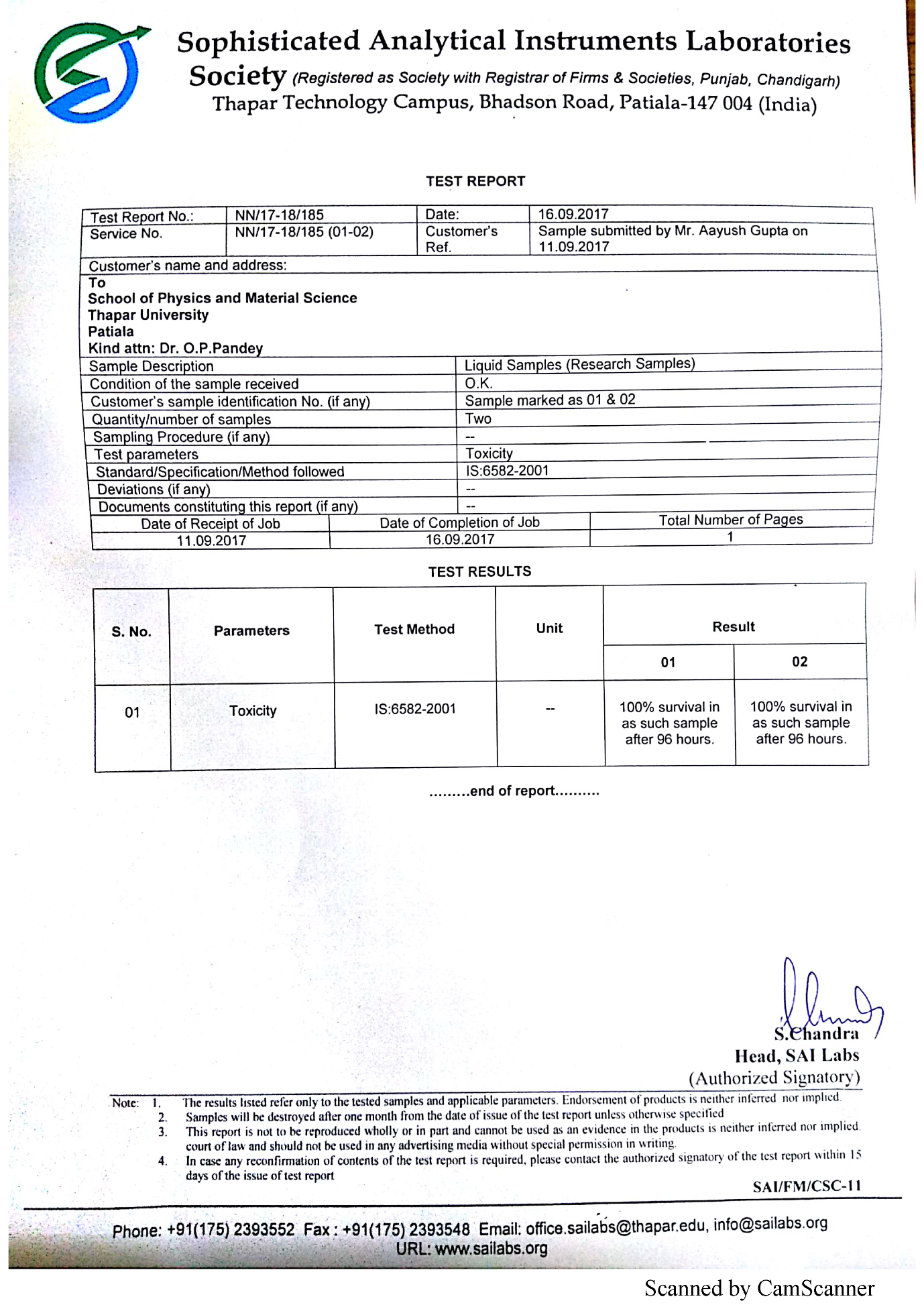


**Figure S-12:** Toxicity test of photodegradaed dye solution by 10C800 (represented as 1) and 5C800 (represented as 2) by observing survival of fish after 96hours.

**Figure S-13:** XRD pattern of reactants used for the synthesis of NbC (a) Nb_2_O_5_ and (b) activated charcoal. The XRD pattern of activated charcoal shows the amorphous nature of carbon with a slight crystalline peak around 26° associated to graphite. Furthermore, the XRD pattern of Nb_2_O_5_ shows the presence of single phase Nb_2_O_5_ without any presence of other oxides of Nb metal and with the help of Scherrer’s method, the crystallite size is calculated as ~55 nm.

**Figure S-14:** XRD patterns of synthesized samples at 800°C for 10 h of holding with different amount of activated charcoal (0.5, 1.0 and 1.5 g). These XRD patterns signify the lack of carbon (0.5 and 1.0 g) for complete carburization of Nb.

 **
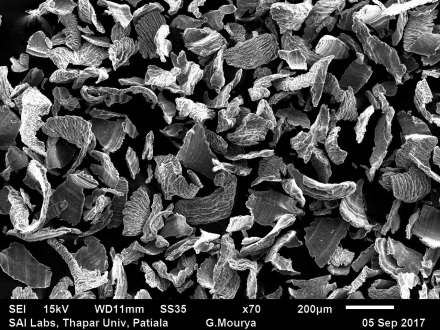
**

**
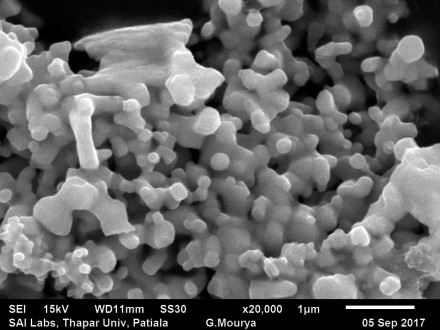
**

**
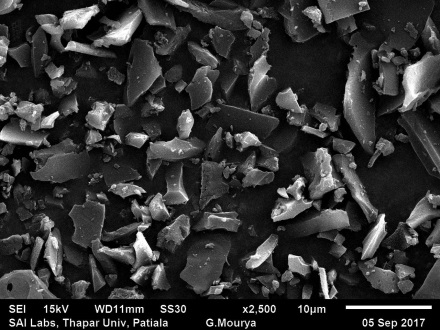
**

**Figure S-15:** Histogram and SEM micrograph of Mg powder, Nb_2_O_5_ and activated charcoal used for the synthesis of carbon coated NbC nanoparticles. With the help of histograms, the average particle size of Mg, Nb_2_O_5_ and activated charcoal comes out to be 167 µm, 257 nm and 4.23 µm, respectively. Further, metallic Mg powder has flake like morphology while activated charcoal possess sharp edged faceted particles. Due to the smaller particle size, agglomerated Nb_2_O_5_ particles were observed.

**Figure S-16:** N_2_ adsorption-desorption behavior of charcoal resulting 51 m^2^/g as specific surface area.

**Appendix-II**

**Williamson-Hall Analysis**

Since the combined effect of instrumental and specimen broadening is recorded in XRD pattern, the instrumental broadening factor has to be eliminated from each reflection by using the following relation;

| $\boldsymbol{\beta}_{\mathbf{hkl}}\mathbf{=}\sqrt{\boldsymbol{\beta}_{\mathbf{measured}}^{\mathbf{2}}\mathbf{-}\boldsymbol{\beta}_{\mathbf{instrumental}}^{\mathbf{2}}}$ | (i) |
| --- | --- |

The size of coherent source of diffraction i.e. crystallite size (t) is estimated with the help of Scherrer formula using ‘2θ’ values obtained from fitted profile. Scherrer proposed the inverse proportional relationship between crystallite size and broadening of Bragg peak (β_hkl_) which is as follows;

| $\boldsymbol{\beta}_{\mathbf{hkl}}\mathbf{=}\frac{\boldsymbol{k\lambda}}{\mathbf{t.cos}\boldsymbol{\theta}_{\mathbf{hkl}}}$ | (ii) |
| --- | --- |

where θ_hkl_: Bragg angle, k: shape factor (0.9), and λ: X-ray wavelength (~0.15406 nm). In contrast to this theory, the contribution of strain in broadening of Bragg peaks was also projected by Wilson and Stokes [3] which suggested the magnitude of induced strain (ε) in crystallite, can be expressed as,

| $\boldsymbol{\beta}_{\mathbf{hkl}}\boldsymbol{= 4\varepsilon.tan}\boldsymbol{\theta}_{\mathbf{hkl}}$ | (iii) |
| --- | --- |

The contribution of crystallite size and strain in peak broadening can be conveyed with the help of following equation which separates out the individual effect and also referred as uniform deformation model (UDM) of Williamson-Hall analysis;

| $\boldsymbol{\beta}_{\mathbf{hkl}}\mathbf{=}\frac{\boldsymbol{k\lambda}}{\mathbf{t.cos}\boldsymbol{\theta}_{\mathbf{hkl}}}\boldsymbol{+}\boldsymbol{4\varepsilon.tan}\boldsymbol{\theta}_{\mathbf{hkl}}$ | (iv) |
| --- | --- |

The above relationship follows the isotropic strain distribution throughout the crystal which provides the magnitude of strain and crystallite size as slope and intercept on ordinate in ‘β_hkl_cosθ_hkl_’ vs. ‘4sinθ_hkl_’ plot respectively. Though homogeneity of strain might not be satisfied in many nanomaterials, isotropic stress and isotropic strain energy density provide more logical explanations for the induced lattice strain [4]. These are referred as uniform stress deformation model (USDM) and uniform strain energy density model (USEDM) respectively and can be described mathematically as follows:

| USDM | $\boldsymbol{\beta}_{\mathbf{hkl}}\mathbf{=}\frac{\boldsymbol{k.\lambda}}{\mathbf{t.cos}\boldsymbol{\theta}_{\mathbf{hkl}}}\mathbf{+}\frac{\boldsymbol{4\sigma.tan}\boldsymbol{\theta}_{\mathbf{hkl}}}{\mathbf{E}_{\mathbf{hkl}}}$ | (v) |
| --- | --- | --- |
| USEDM | $\boldsymbol{\beta}_{\mathbf{hkl}}\mathbf{=}\frac{\boldsymbol{k.\lambda}}{\mathbf{t.cos}\boldsymbol{\theta}_{\mathbf{hkl}}}\mathbf{+4tan}\boldsymbol{\theta}_{\mathbf{hkl}}\left( \frac{\mathbf{2u}}{\mathbf{E}_{\mathbf{hkl}}} \right)^{\mathbf{0.5}}$ | (vi) |

where $E_{hkl}^{-1}=S_{11}-2\left( S_{11}-S_{12}-\frac{S_{44}}{2} \right)\left( l^{2}m^{2}+m^{2}n^{2}+n^{2}l^{2} \right)$ [5], S_11_= 1.7826×10^-3^ GPa^-1^, S_12_= -0.3913×10^-3^ GPa^-1^ and S_44_= 7.1428×10^-3^ GPa^-1^ [6]. USDM and USEDM assume the homogeneity of stress and strain energy density to calculate the induced strain by hook’s law (σ_hkl_ = E_hkl_.ε_hkl_ and u = ε^2^E_hkl_/2) respectively which are shown in **Figure S-17**. tanθ_hkl_, instead of (cosθ_hkl_)^-1^ affects the magnitude of β_hkl_ by separating the diffracted beams corresponding to combination of crystallite size and strain [7]. XRD profiles provide information on crystallite size and distortion within the crystallites for which line profile criteria of Pearson’s VII function were used to calculate the position of the Bragg peak (2θ) and full width at half maxima (FWHM). Pearson’s VII function assumes the symmetric nature of peaks about peak position and provides more realistic results than Gaussian and Lorentzian function due to the dependency of Pearson’s VII on both Gaussian and Lorentzian functions [8]. The best results of fitted line profile were taken into account with minimal error and a sample of fitted line profile of Bragg peak of (111) planes of sample 10C800 is shown in **Figure** **S-17a**. With the help of fitted profiles of XRD peaks of NbC, the amount of strain constituted within the lattice has been analyzed by Williamson-Hall (W-H) analyses. Basic assumptions and mathematical models of W-H analysis are explained in Appendix-II (Supplementary information). Linear fitting and postulates of various models of W-H analysis are shown in **Figure** **S-17 (b-d)** and **Table 3** (main manuscript) respectively.

| **** | **** |
| --- | --- |
| **** | **** |

**Figure S-17:** Williamson-Hall analyses of sample 10C800; (a) Pearson’s VII fitting of peak (111), (b) USM model, (c) USDM model and (d) USDEM model.

**Proposed synthesis mechanism:**

|  | $\mathrm{Nb}_{2}O_{5}+Mg\to2NbO_{2}+MgO$ |  |
| --- | --- | --- |
|  | $\mathrm{Nb}_{2}O_{5}+C\to2NbO_{2}+CO$ |  |
|  | $\mathrm{Nb}_{2}O_{5}+CO\to2NbO_{2}+CO_{2}$ |  |
|  | ${2Nb}_{2}O_{5}+C\to4NbO_{2}+CO_{2}$ |  |
|  | ${2Nb}_{2}O_{5}+C+Mg\to4NbO_{2}+MgO+CO$ |  |
|  | ${3Nb}_{2}O_{5}+C+Mg\to6NbO_{2}+MgO+CO_{2}$ |  |
|  | ${2Nb}_{2}O_{5}+CO+Mg\to4NbO_{2}+MgO+CO_{2}$ |  |
|  | ${4Nb}_{2}O_{5}+C+CO+Mg\to8NbO_{2}+MgO+2CO_{2}$ |  |
|  | $\mathrm{Nb}_{2}O_{5}+3Mg\to2NbO+3MgO$ |  |
|  | $\mathrm{Nb}_{2}O_{5}+3C\to2NbO+3CO$ |  |
|  | $\mathrm{Nb}_{2}O_{5}+3CO\to2NbO+3CO_{2}$ |  |
|  | ${2Nb}_{2}O_{5}+3C\to4NbO+3CO_{2}$ |  |
|  | $\mathrm{Nb}_{2}O_{5}+C+2Mg\to2NbO+2MgO+CO$ |  |
|  | $\mathrm{Nb}_{2}O_{5}+C+Mg\to2NbO+MgO+CO_{2}$ |  |
|  | $\mathrm{Nb}_{2}O_{5}+CO+2Mg\to2NbO+2MgO+CO_{2}$ |  |
|  | ${2Nb}_{2}O_{5}+C+2CO+2Mg\to4NbO+2MgO+2CO_{2}$ |  |
|  | $\mathrm{Nb}_{2}O_{5}+2Mg\to NbO_{2}+NbO+2MgO$ |  |
|  | $\mathrm{Nb}_{2}O_{5}+2C\to NbO_{2}+NbO+ 2CO$ |  |
|  | $\mathrm{Nb}_{2}O_{5}+2CO\to NbO_{2}+NbO+2CO_{2}$ |  |
|  | $\mathrm{Nb}_{2}O_{5}+C\to NbO_{2}+NbO+CO_{2}$ |  |
|  | $\mathrm{Nb}_{2}O_{5}+C+Mg\to NbO_{2}+NbO+MgO+CO$ |  |
|  | ${2Nb}_{2}O_{5}+C+Mg\to2NbO_{2}+2NbO+2MgO+CO_{2}$ |  |
|  | $\mathrm{Nb}_{2}O_{5}+CO+Mg\to NbO_{2}+NbO+MgO+CO_{2}$ |  |
|  | $2\mathrm{Nb}_{2}O_{5}+C+2CO+Mg\to NbO_{2}+3NbO+MgO+3CO_{2}$ |  |
|  | $\mathrm{Nb}O_{2}+C+2Mg\to NbC+2MgO$ |  |
|  | $\mathrm{Nb}O_{2}+2C+Mg\to NbC+MgO+CO$ |  |
|  | $2NbO_{2}+3C+2Mg\to2NbC+2MgO+CO_{2}$ |  |
|  | $2NbO_{2}+4C+Mg\to2NbC+MgO+CO+CO_{2}$ |  |
|  | $\mathrm{Nb}O_{2}+2CO+2Mg\to NbC+2MgO+CO_{2}$ |  |
|  | $\mathrm{Nb}O_{2}+C+CO+Mg\to NbC+MgO+CO_{2}$ |  |
|  | $NbO+C+Mg\to NbC+MgO$ |  |
|  | $2NbO+3C+Mg\to2NbC+MgO+CO$ |  |
|  | $4NbO+6C+Mg\to4NbC+MgO+CO+CO_{2}$ |  |
|  | $NbO+2CO+Mg\to NbC+MgO+CO_{2}$ |  |
|  | $2NbO+2C+CO+Mg\to2NbC+MgO+CO_{2}$ |  |
|  | $3NbO+4C+Mg\to3NbC+MgO+CO_{2}$ |  |
|  | $\mathrm{Nb}O_{2}+NbO+2C+3Mg\to2NbC+3MgO$ |  |
|  | $\mathrm{Nb}O_{2}+NbO+3C+2Mg\to2NbC+2MgO+CO$ |  |
|  | $\mathrm{Nb}O_{2}+NbO+3C+Mg\to2NbC+MgO+CO_{2}$ |  |
|  | $\mathrm{Nb}O_{2}+2NbO+5C+Mg\to3NbC+MgO+CO+CO_{2}$ |  |
|  | $\mathrm{Nb}O_{2}+NbO+4CO+3Mg\to2NbC+3MgO+2CO_{2}$ |  |
|  | $\mathrm{Nb}O_{2}+NbO+2C+CO+2Mg\to2NbC+2MgO+CO_{2}$ |  |

For the reduction of Nb_2_O_5_, the values of ΔHRR_f_RR at standard pressure corresponding to the above chemical reactions were estimated by

| $\boldsymbol{\Delta}\boldsymbol{G}_{\boldsymbol{T}}\mathbf{=}\boldsymbol{\Delta}\boldsymbol{H}_{\mathbf{0}}\mathbf{+}\int_{\boldsymbol{T}_{\mathbf{0}}}^{\boldsymbol{T}} \boldsymbol{C}_{\boldsymbol{P}}\boldsymbol{dT}\mathbf{-}\boldsymbol{T}\left[ \boldsymbol{\Delta}\boldsymbol{S}_{\mathbf{0}}\mathbf{+}\int_{\boldsymbol{T}_{\mathbf{0}}}^{\boldsymbol{T}} \frac{\boldsymbol{C}_{\boldsymbol{P}}}{\boldsymbol{T}}\boldsymbol{dT} \right]$**,** $\boldsymbol{C}_{\boldsymbol{p}}\boldsymbol{= \Delta}\boldsymbol{a}\boldsymbol{+ \Delta}\boldsymbol{b}\boldsymbol{.\Delta}\boldsymbol{T}\boldsymbol{+ \Delta}\boldsymbol{c}\boldsymbol{.\Delta}\boldsymbol{T}^{\mathbf{-2}}$ | (lx) |
| --- | --- |

where, (Δa, Δb, Δc), ΔH_0_ and ΔS_0_ are the difference of the coefficient of heat capacities, enthalpy and entropy at 298K of products and reactants, respectively. The negative values of Gibbs free energy (ΔG) for the above-mentioned reactions convey the feasibility of the reaction as shown in **Figure 6**.

**References:**

1. Z.Q. Li, H.F. Zhang, X.B. Zhang, Y.Q. Wang, X.J. Wu, Nanocrystalline tungsten carbide encapsulated in carbon shells, Nanostruct. Mater. 10 (1998) 179-184.
2. A. Gupta, G. Singla, O.P. Pandey, Effect of synthesis parameters on structural and thermal properties of NbC/C nanocomposite synthesized via in-situ carburization reduction route at low temperature, Ceram. Inter. 42 (2016) 13024-13034.
3. S. Vives, E. Gaffet, C. Meunier, X-ray diffraction line profile analysis of iron ball milled powders, Mater. Sci. Eng. A 366 (2004) 229-238.
4. Y. Rosenberg, V.S. Machavariant, A. Voronel, S. Garber, A. Rubshtein, A.I. Frenkel, E.A. Stern, Strain energy density in the X-ray powder diffraction from mixed crystals and alloys, J. Phys. Condens. Matter 12 (2012) 8081-8088.
5. E.A. Brands, G.B. Brook, (Eds), In Smithells Metals Reference Book, Butterworth-Heinemann, Linacre House, Jordan Hill, Oxford (1999).
6. J. Chen, L.L. Boyer, H. Krakauer, M.J. Mehl, Elastic constants of NbC and MoN: Instability of B_1_-MoN, Phys. Rev. B 37 (1988) 3295-3298.
7. A.K. Zak, W.H. Abd Majid, M.E. Abrishami, R. Yousefi, X-ray analysis of ZnO nanoparticles by Williamson–Hall and size–strain plot methods, Solid State Sci. 13 (2011) 251-256.
8. (ed.) E. Prince, International tables for crystallography – mathematical, physical and chemical tables, KLUWER academic publishers, Boston, London (2004).
